# The structure of tyrosine-10 favors ionic conductance of Alzheimer’s disease-associated full-length amyloid-β channels

**DOI:** 10.1038/s41467-023-43821-y

**Published:** 2024-02-13

**Authors:** Abhijith G. Karkisaval, Rowan Hassan, Andrew Nguyen, Benjamin Balster, Faisal Abedin, Ratnesh Lal, Suren A. Tatulian

**Affiliations:** 1 Department of Mechanical and Aerospace Engineering, University of California San Diego, La Jolla, CA USA; 2 Department of Physics, University of Central Florida, Orlando, FL USA; 3 Department of Bioengineering, University of California San Diego, La Jolla, CA USA; 4 Present Address: Department of Biology, Xavier University of Louisiana, New Orleans, LA USA

**Keywords:** Alzheimer's disease, Biological physics, Ion channels in the nervous system

## Abstract

Amyloid β (Aβ) ion channels destabilize cellular ionic homeostasis, which contributes to neurotoxicity in Alzheimer’s disease. The relative roles of various Aβ isoforms are poorly understood. We use bilayer electrophysiology, AFM imaging, circular dichroism, FTIR and fluorescence spectroscopy to characterize channel activities of four most prevalent Aβ peptides, Aβ_1-42_, Aβ_1-40_, and their pyroglutamylated forms (AβpE_3-42_, AβpE_3-40_) and correlate them with the peptides’ structural features. Solvent-induced fluorescence splitting of tyrosine-10 is discovered and used to assess the sequestration from the solvent and membrane insertion. Aβ_1-42_ effectively embeds in lipid membranes, contains large fraction of β-sheet in a β-barrel-like structure, forms multi-subunit pores in membranes, and displays well-defined ion channel features. In contrast, the other peptides are partially solvent-exposed, contain minimal β-sheet structure, form less-ordered assemblies, and produce irregular ionic currents. These findings illuminate the structural basis of Aβ neurotoxicity through membrane permeabilization and may help develop therapies that target Aβ-membrane interactions.

## Abstract (web summary)

The structural basis of membrane permeabilization by Alzheimer’s disease-related amyloid β (Aβ) peptides is elucidated. Membrane insertion of tyrosine-10 supports the most effective ionic conductance of the full-length Aβ_1-42_ compared to other isoforms.

## Introduction

Aberrant fibrillar deposits of the amyloid β (Aβ) peptide constitute a major histopathological trait of Alzheimer’s disease (AD) brains^1,2^. There is compelling evidence that the soluble oligomers of Aβ exert the main neurotoxic effect^3–5^. Aβ occurs in different isoforms; while the 42- and 40-residue peptides (Aβ_1-42_ and Aβ_1-40_) are the most abundant species, shorter forms resulting from proteolysis are also present^6^. Of particular interest are the N-terminally truncated and pyroglutamylated Aβ peptides (AβpE) as they are present in AD brains in substantial amounts (up to 25% of total Aβ) and are hypertoxic^7–10^. The augmented cytotoxicity of AβpE has been attributed to elevated β-sheet formation and fibrillogenesis propensity^7,11–13^. However, other publications have shown similar β-sheet structure and slower fibril formation by AβpE species as compared to their unmodified counterparts^14–17^.

The molecular mechanisms of Aβ neurotoxicity is complex and not fully understood. Aβ can damage neurons through dysregulation of cellular ionic homeostasis by two main routes: (i) modulation of certain ion channels in plasma or intracellular membranes^18,19^ or (ii) permeabilization of cell membranes through direct membrane insertion and ion channel formation^7,20–33^. Aβ_1-42_ and Aβ_1-40_ induced ion conductance in lipid membranes^20,27,34^. Patch clamp experiments and optical imaging of ion channel conductance on neurons identified cation-selective, Zn^2+^-sensitive channels induced by Aβ_1-42_ and Aβ_1-40_, much like those observed in lipid bilayers^21,22,27,35^. Significantly, channel activity of Aβ_1-42_ was stronger in the membranes prepared using brain extract lipids^28^. Moreover, these effects have been eliminated by peptides or small molecules that specifically block Aβ channels^5,23–25^, inhibit Aβ aggregation and membrane insertion^29^, or competitively prevent the binding of Aβ to membrane lipids^36^. In HEK293 cells, Aβ_1-42_ oligomers formed ion channels but Aβ_1-40_ did not show channel activity in any aggregation state^30^. The inability of Aβ_1-40_ to form channels in cell membranes echoes with the absence of electrical activity of lipid membranes upon addition of micelle-solubilized Aβ_1-40_, in contrast to Aβ_1-42_^37^, or nonspecific membrane perturbation by Aβ_1-40_ through binding to the bilayer surface^38^.

Membrane pore formation and membranotropic neurotoxicity of AβpE has been studied less extensively. AβpE_3-42_ oligomers were shown to insert into anionic lipid membranes and form Zn^2+^-sensitive channels more efficiently than Aβ_1-42_^39,40^. AβpE_3-42_ also allowed higher Ca^2+^ influx into cultured mouse cortical neurons than the unmodified Aβ_1-42_ peptide^7^. Membrane channel data for AβpE_3-40_ have not been reported to the best of our knowledge.

Structures of Aβ peptides have been examined by computational and experimental methods. Aβ_1-40_ channels have been simulated based on secondary structure predictions as annular hexameric or dodecameric assemblies where the pore is lined by the polar face of an N-terminal amphipathic β-hairpin while the C-terminal hydrophobic α-helix is in contact with membrane lipids^41,42^. Atomic force microscopy (AFM) images of lipid membranes with reconstituted Aβ_1-42_ identified channel-like structures composed of four or six subunits with an overall outer diameter of 8–12 nm^20^. Meanwhile, spectroscopic studies revealed β-sheet-rich secondary structure for membrane-embedded Aβ_1-40_ and Aβ_1-42_^38,43^. Based on this information and NMR-derived structures of Aβ_1-40_ and Aβ_1-42_ fibrils^44–46^, molecular dynamics (MD) simulations produced structural models for channel-forming Aβ fragments N9 and p3 where 12–36 monomers form annular structures composed of loosely connected subunits, each containing 3-7 H-bonded U-shaped strand-turn-strand motifs with N- and C-terminal strands oriented inward and outward, respectively^4,47,48^. Similar structures of Aβ_1-42_ were modeled where six antiparallel β-barrels assemble to form a hexamer of hexamers or rearrange into one annular structure providing a transmembrane pore lined by the N-terminal polar residues^49^. Another Aβ_1-42_ pore model was proposed as a tetramer or hexamer of transmembrane 2-stranded β-sheets upstream of residue 17 with N-terminal β-hairpins extending into the aqueous phase^50^. Further MD studies generated a β-barrel pore model for AβpE_3-42_ that comprised 18 tilted transmembrane U-shaped structures (residues 11-15 to 42) with an unordered N-terminus tending to insert into the membrane due to the apolar terminal lactam ring^39^. The β-barrel or β-sandwich-like structure was confirmed for Aβ_1-42_ in detergent micelles and detergent/lipid bicelles by solution NMR^37,51^. More details on the structural aspects of channel/pore formation by various Aβ species can be found in a recent review by Viles^32^.

The above discussion underscores the paucity or absence of experimentally determined molecular structures of membrane-embedded Aβ species that play central role in AD etiology, i.e., Aβ_1-42_, Aβ_1-40_, AβpE_3-42_, AβpE_3-40_. Combined with inconsistent data or absence of data on ion channel formation by some important forms of Aβ, e.g., Aβ_1-40_ and AβpE_3-40_, it is imperative to undertake a systematic analysis of channel formation abilities and structures of these peptides reconstituted in lipid bilayers. Here we report previously uncharacterized relationship between the molecular structure and morphology of four Aβ isoforms, namely, Aβ_1-42_, Aβ_1-40_, AβpE_3-42_, AβpE_3-40_ and their ion channel activities. Aβ_1-42_ efficiently embeds in membranes, displays maximum β-sheet structure, forms multi-subunit annular assemblies in membranes, and induces step-like single channels. The other three peptides display less effective membrane insertion, smaller β-sheet content, form irregular supramolecular assembles and cause current spikes or bursts of relatively large conductance. Identification of the structural features that support certain types of membrane currents by various Aβ species enhance our understanding of the molecular basis for membrane permeabilization and cytotoxicity of the most important Aβ peptides involved AD etiology.

## Results

### Membrane channel forming activities of the peptides

**Fig. 1:**
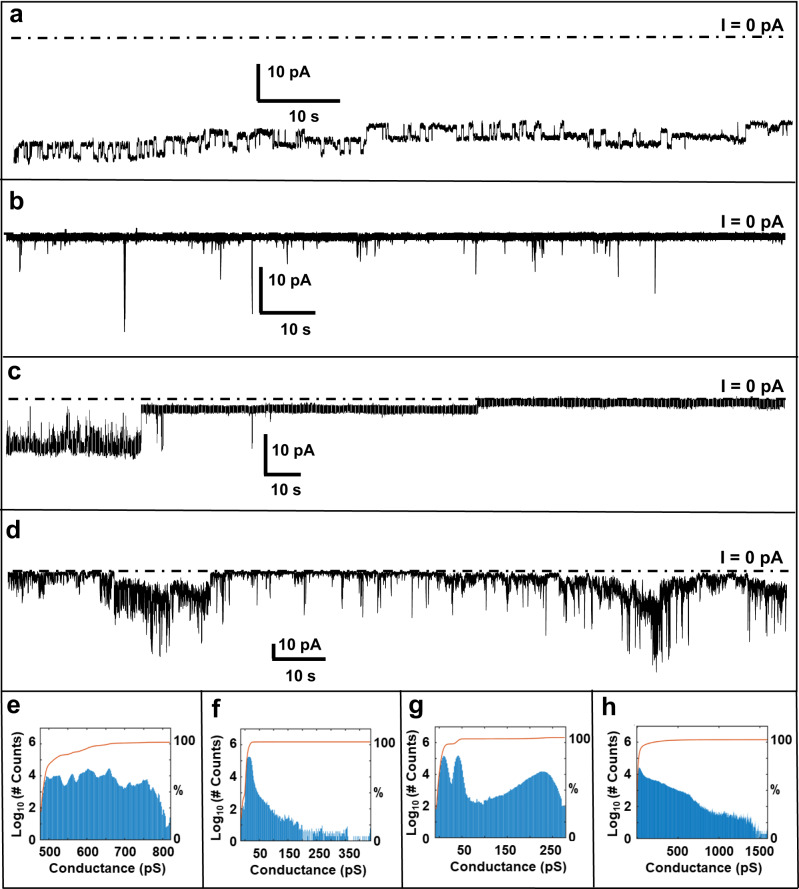
Voltage clamp current traces and conductance histograms. Aβ_1-42_ (**a**), Aβ_1-40_ (**b**), AβpE_3-42_ (**c**), and AβpE_3-40_ (**d**) at 50 mV membrane-hold potential in 1 M KCl + 10 mM HEPES buffer, pH 7.4. The membranes were composed of 1-palmitoyl-2-oleoyl-phosphatidylcholine (POPC), 1-palmitoyl-2-oleoyl-phosphatidyglycerol (POPG), and cholesterol (6:3:1 molar ratio). The dotted horizontal line indicates the zero current baseline level. Conductance histograms for Aβ_1-42_, Aβ_1-40_, AβpE_3-42_, and AβpE_3-40_ are shown in (**e**–**h**) respectively, where the total counts/bin are expressed in log scale in the left y-axis (blue bars) and the cumulative counts (0–100%) per conductance level are indicated in the right y-axis (red line). At least five independent experiments have been conducted with similar results.

Voltage clamp experiments have been conducted at selected hold voltages between +100 mV and −100 mV (the sign corresponds to the trans side, where the peptide was added). At +50 mV transmembrane voltage, the current reached a baseline level of ~40 pA at ~5 min following addition of Aβ_1-42_, featuring stepwise transitions between discrete conductance levels (Fig. 1a). The stability of the conductance pattern and the consistent amplitude of the transitions suggest that Aβ_1-42_ inserts into the membrane and forms ion-conducting channels that are able to switch between on/off states.

A similar behavior of stepwise current transitions was observed for Aβ_1-42_ at other voltages, although the step-like pattern was superimposed with gradual changes of the total conductance level, possibly indicating the presence of a heterogeneous set of ion-conducting structures such as assemblies of different oligomeric numbers (Supplementary Fig. 1), summarized in the histogram (Fig. 1e).

Aβ_1-40_ displayed a different behavior; infrequent burst-like current spikes with <50 ms duration were detected that were superimposed on a zero current level (Fig. 1b). The amplitude, duration, and the frequency of current spikes were significantly higher at 100 mV and -100 mV applied voltages, more frequent at -100 mV than +100 mV, and their magnitude exceeded severalfold the current steps induced by Aβ_1-42_ (Supplementary Fig. 2). Non-zero macro current was not observed for this peptide. The conductance histogram of Aβ_1-40_ featured a peak around the zero level and a relatively wide range for conductance distribution (Fig. 1f).

Membrane conductance induced by AβpE_3-42_ involved both stepwise and high frequency burst-like patterns (Fig. 1c, Supplementary Fig. 3). In addition, events of sudden jump between different macro conductance levels occurred, which might result from cooperative opening/closing of multiple channels (Supplementary Fig. 3). Some unique patterns of AβpE_3-42_ were the presence of constant macro conductance levels for long dwell time (~1 min) as well as shifts to zero current level, both of which could be overlayed with short bursts of current. This behavior is reflected in the distinct bimodal conductance histogram of this peptide (Fig. 1g, Supplementary Fig. 3).

AβpE_3-40_ also exhibited a combination of step-like and burst-like activities, with higher frequency and higher conductance compared to Aβ_1-40_ and AβpE_3-42_ (Fig. 1d). The burst-like activities of AβpE_3-40_ were continuous throughout the recording and showed similar behavior at all tested voltages. The conductance histogram of AβpE_3-40_ was spread over a range of values, with no clearly discernible peaks (Fig. 1h, Supplementary Fig. 4). Even though clear step-like transitions in the conductance were observed for some voltages, rapid switching between sub-states of intermittent conductance also occurred. The change in voltage did not alter the kinetic behavior of the channels, and the frequency of current transition events remained constant.

### Single channel Properties

**Fig. 2:**
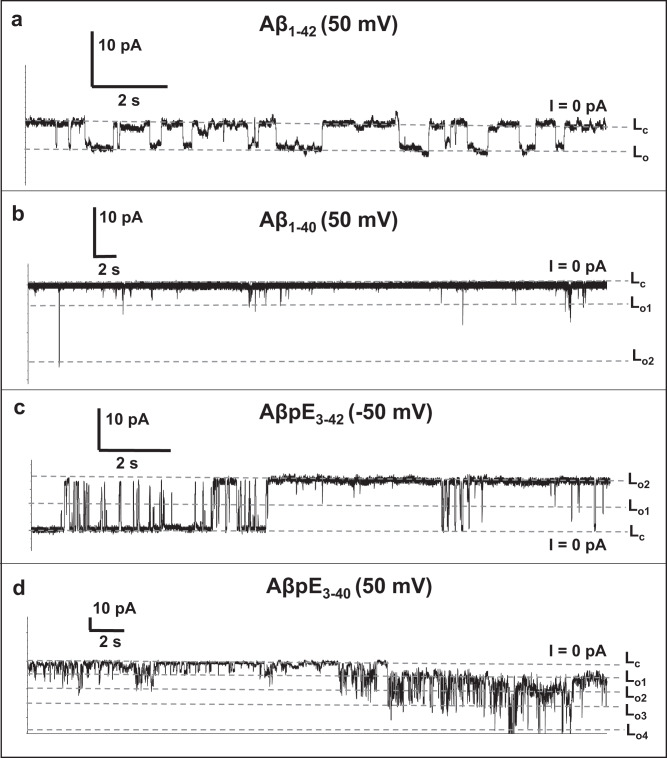
Electrophysiological recordings. Current traces for Aβ_1-42_ (**a**), Aβ_1-40_ (**b**), AβpE_3-42_ (**c**), and AβpE_3-40_ (**d**) in shorter time scale reveal the single channel conductance behaviors. Discrete conductance levels have been marked with notation L_c_, i.e. closed state, and L_o1_, L_o2_, …, corresponding to multiple open state levels. These levels have been assigned to include maximum number of points at a particular conductance level. In all cases except panel **c** (AβpE_3-42_), the hold voltage was +50 mV. For AβpE_3-42_, -50 mV was chosen as the step-like conductance activity was more distinguishable compared to the +50 mV trace. Membrane lipid composition was the same as in Fig. 1, and the buffer was 1 M KCl + 10 mM HEPES, pH 7.4. At least five independent experiments have been conducted with similar results.

A close-up view of current traces revealed the single channel properties. Aβ_1-42_ channels switched between closed and open states, designated L_c_ and L_o_, respectively, with channel conductance of ~100 pS (Fig. 2a). The open state dwell time varied in a wide range, from < 100 ms to >1 s, indicating slow on/off kinetics. The other peptides displayed more than one open sub-state, designated L_o1_, L_o2_, etc. (Fig. 2). Aβ_1-40_ mostly exhibited burst-like activity, i.e., jumps from L_c_ to a short-lived L_o1_ state (80-100 pS) and back, although other states of larger conductance ( ~340 pS) are possible (Fig. 2b). Thus, Aβ_1-40_ does not form stable channels with significant open state dwell time under these conditions.

AβpE_3-42_ showed regular switching between discrete states L_c_ and L_o2_ (~300 pS) while also transitioning to a more seldom intermediate states L_o1_ (~150 pS) (Fig. 2c). This peptide showed relatively fast kinetics of transitions between states in the beginning of the trace followed by periods of very long (>1 s) open state dwell time, resembling the behavior of Aβ_1-42_. AβpE_3-40_ displayed a complex behavior, i.e., twitching between at least 4 open states (L_o1_ through L_o4_) with conductance values of ~200 pS, 400 pS, 640 pS and 1040 pS, respectively (Fig. 2d). In contrast to the other three peptides, AβpE_3-40_ showed continuously transitioning events with relatively fast kinetics.

### Channel Blocking by Zn^2+^ Ions

Since Zn^2+^ ions block Aβ-induced membrane currents (see Introduction), the ability of Zn^2+^ to modulate Aβ channels has been studied. At +100 mV hold voltage, Aβ_1-42_ induced current of ~65 pA, followed by single channel activity (Supplementary Fig. 5a). After first addition of 10 mM ZnCl_2_, the single channel activity persisted for roughly 40 s, then ceased as the membrane switched to a lower macro conductance level. The current did not fully subside even after a second addition of 10 mM ZnCl_2_, although most of the flickering activity stopped. Thus, Zn^2+^ does exert an inhibitory effect on Aβ_1-42_ channels but a fraction of ion-conducting structures remains active, consistent with a heterogeneous assembly of Aβ_1-42_ molecules in the membrane.

Aβ_1-40_ showed the characteristic burst-like activity, which ceased ~30 s after addition of 10 mM ZnCl_2_, followed by an unusual behavior of the current, i.e., appearance of long-lived current flickers (or closely spaced short bursts) in addition to infrequent single bursts. This “anomalous” behavior of the current trace has only been seen for Aβ_1-40_ but neither for the other three peptides nor for membranes without peptide addition. Hence, it reflects the unique structural and membrane interaction properties of Aβ_1-40_, as discussed in the forthcoming sections. At the end of the trace, the current vanished, hence no more ZnCl_2_ was added (Supplementary Fig. 5b).

AβpE_3-42_ showed large macro conductance, which subsided significantly with the first addition of 10 mM ZnCl_2_. Following a second addition of ZnCl_2_, the residual conductance persisted for ~4 min and then stopped, with current returning to the zero baseline level (Supplementary Fig. 5c). AβpE_3-40_ displayed continuous, high frequency burst-like activity (Supplementary Fig. 5d). The first 10 mM ZnCl_2_ addition reduced the amplitude of the spikes, but higher conductance resumed after ~3 min. Approximately 4 min after second addition of 10 mM ZnCl_2_, the current died out.

### Peptide Structure from Circular Dichroism

**Fig. 3:**
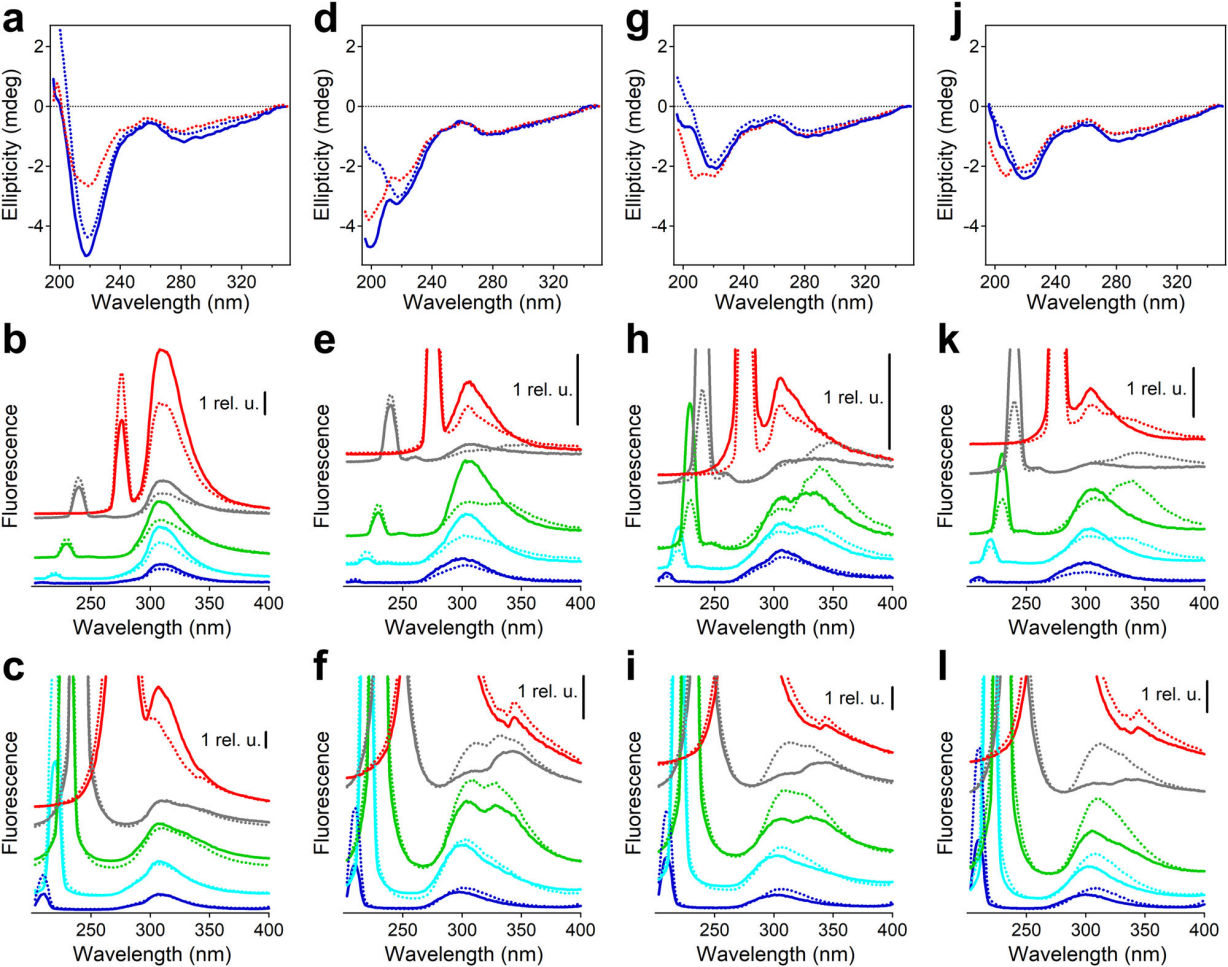
CD and fluorescence spectra. Aβ_1-42_ (**a**–**c**), Aβ_1-40_ (**d**–**f**), AβpE_3-42_ (**g**–**i**), and AβpE_3-40_ (**j**–**l**) with or without lipid. In CD spectra (upper row), blue and red lines correspond to the absence and presence of lipid vesicles, respectively (POPC:POPG:cholesterol at 6:3:1 molar ratio). For fluorescence data, the middle row presents spectra in the absence of lipid and the lower row shows data in the presence of lipid vesicles. The excitation was at 210 nm (blue), 220 nm (cyan), 230 nm (green), 240 nm (gray), and 275 nm (red). Dotted lines indicate that the samples have been extruded through 100 nm pore-size polycarbonate membranes for both CD and fluorescence spectra. Peptide and total lipid (when present) concentrations were 20 μM and 1 mM, respectively, the buffer was 25 mM NaCl + 25 mM Na,K-phosphate, pH 7.2, and the temperature was 20^o^C. Three independent experiments have been conducted with similar results.

Significant differences in the channel activities of the four Aβ peptides are likely due to their distinct structural features and modes of interactions with lipid membranes. The secondary structure of the peptides without and with lipid vesicles was probed by far-UV circular dichroism (CD). Because peptide samples with lipid vesicles were studied before and after extrusion through 100 nm pore-size filters to obtain unilamellar vesicles of defined size, lipid-free peptide samples were also studied before and after extrusion. Spectra of Aβ_1-42_ displayed a deep minimum at 217-218 nm irrespective of extrusion, indicating β-sheet structure^52^ (Fig. 3a). Extruded samples with lipid showed a weaker minimum at 216 nm and a shoulder around 209 nm, suggesting an α/β-type structure. (Unextruded samples with lipid generated spectra with excessive noise due to strong light scattering by vesicles and therefore are not shown.) Aβ_1-40_ generated minima around 217 and 199 nm before extrusion, implying β-sheet and unordered structures (Fig. 3d), and after extrusion the spectrum displayed one minimum at 217 nm assigned to β-sheet. In contrast to Aβ_1-42_, the presence of lipid caused formation of unordered (rather than α-helical) structure in Aβ_1-40_. Spectra of AβpE_3-42_ and AβpE_3-40_ showed weaker and red-shifted nπ* transitions (219-222 nm) before and after extrusion (Fig. 3g, j). With lipid vesicles, spectra of these peptides exhibited an additional feature around 208 nm, indicating α-helix formation.

Thus, Aβ_1-42_ forms well-defined β-sheet structure, followed by the pyroglutamylated peptides, while Aβ_1-40_ tends to be more unordered. In all cases except Aβ_1-40_, the presence of membranes appears to induce partial α-helical structure. Together with the channel data, these results indicate a correlation between β-sheet propensity and step-like channel forming potency.

In the near-UV region, the CD spectra displayed a negative band at 278-282 nm, which is the induced CD signal due to the S_0_→S_1_ (ground to first excited singlet state) transition in the aromatic amino acid side chains^53^. This CD band did not depend on the presence of lipid. However, fluorescence spectroscopy revealed interesting structural features of the peptides, described in the next section.

### Structural features from fluorescence spectroscopy

As described in Supplementary Fig. 6, excitation of Aβ_1-42_ at wavelength (λ_ex_) varying from 200 nm to 380 nm results in two effects, Rayleigh scattering at λ_ex_ and fluorescence at two regions of λ_ex_, i.e., 220-230 nm and 260-280 nm, due to S_0_→S_2_ and S_0_→S_1_ transitions, respectively. These transitions are also known as ^1^L_a_ and ^1^L_b_, respectively, according to Platt designation^54^ and are schematically illustrated in Supplementary Fig. 7. Aβ_1-42_ produced strong tyrosine (Tyr) fluorescence at 307-310 nm with λ_ex_ varying between 210 nm and 275 nm before and after extrusion (Fig. 3b). Similar emission wavelengths following S_0_→S_2_ and S_0_→S_1_ excitations indicate S_2_→S_1_ internal conversion and emission from a low vibrational energy state of S_1_ in both cases, consistent with Kasha’s rule (see Supplementary Fig. 7). Aβ_1-40_ displayed weaker and blue-shifted fluorescence (301-307 nm), and the blue shift was stronger at shorter λ_ex_ (Fig. 3e). These features suggest that phenylalanine (Phe) contributes to fluorescence more than in case of Aβ_1-42_ because Tyr of Aβ_1-40_ is exposed to and quenched by the buffer^55^. For the extruded samples of Aβ_1-40_, an interesting effect has been detected. At λ_ex_ = 230 nm, the emission band was split into peaks around 306 and 340 nm, and with λ_ex_ = 240 nm the new, red-shifted component extended to larger wavelengths. The red-shifted component of Tyr emission can be rationalized in terms of a specific solvent effect, i.e., strong H-bonding of the phenolic OH group to a base such as HPO_4_^2-^ and, possibly, deprotonation. Extrusion rendered a large fraction of the Tyr of Aβ_1-40_ exposed to the buffer, resulting in red-shifted emission. The lack of such splitting in case of Aβ_1-42_ indicates this peptide forms a more compact, solvent-protected β-sheet structure before and after extrusion.

Spectra of AβpE_3-42_ were split, with the red-shifted component around 340 nm (for λ_ex_ = 220 nm and 230 nm) or 350 nm (for λ_ex_ = 240 nm) in addition to the lower-wavelength component at 306-310 nm, and the effect was more pronounced for the extruded samples (Fig. 3h). AβpE_3-40_ generated one emission band at 300-308 nm before extrusion and split bands after extrusion, with a red-shifter component at 333-342 nm (Fig. 3k). The absence of the red-shifted emission at λ_ex_ = 275 nm indicates that the S_0_→S_1_ (^1^L_b_) transition is less solvent-sensitive than the S_0_→S_2_ (^1^L_a_) transition because their dipoles are oriented across and along the phenolic ring, respectively, the latter involving the polar hydroxyl group^55^, as shown in Supplementary Fig. 7. Thus, the whole process where deprotonation is involved is thought to proceed as follows: S_0_→S_2_ transition, excited state deprotonation facilitated by a proton acceptor, S_2_→S_1_ internal conversion, vibrational decay to the lowest energy level of S_1_, emission, i.e. radiative transition to a vibrational level of S_0_. These data indicate that (a) splitting of Tyr fluorescence can be used to assess solvent exposure, and hence the compactness of the tertiary fold, of proteins, (b) fluorescence of solvent-exposed and protected fractions can be excited selectively, and (c) the Tyr_10_ residue is most solvent-exposed in AβpE_3-42_ and most solvent-protected in Aβ_1-42_, the other two peptides showing intermediate solvent exposure of Tyr_10_.

The presence of lipid vesicles resulted in much stronger Rayleigh scattering (Fig. 3c, f, i, l). Fluorescence spectra of Aβ_1-42_ did not undergo splitting (Fig. 3c), as in the absence of lipid, indicating a compact tertiary fold. In case of Aβ_1-40_, the emission was split at λ_ex_ = 230 nm and 240 nm both before and after extrusion (a red-shifted component at 330-344 nm in addition to one at 304-313 nm) (Fig. 3f). This means a solvent-exposed Tyr of Aβ_1-40_ even in the presence of vesicles. A poor membrane insertion ability of Aβ_1-40_ is consistent with its less efficient channel forming activity, as discussed above. A solvent-accessible structure in the presence of lipid before and after extrusion was also exhibited by AβpE_3-42_, although in this case the red-shifted component was less intense and was absent at λ_ex_ = 220 nm (Fig. 3i), suggesting partial membrane insertion. Spectra of AβpE_3-40_ in the presence of lipid did not show splitting, especially for the extruded samples (Fig. 3l), implying a more efficient protection from the solvent by the membranes. The fluorescence of Aβ_1-40_, AβpE_3-42_, and AβpE_3-40_ with excitation at 275 nm was obscured by strong Rayleigh scattering; the feature around 344 nm is most likely the resonance-Raman scattering peak (Fig. 3f, i, l). This underscores the usefulness of using a lower wavelength excitation as it not only clears the spectral window of Tyr emission but also reveals the degree of solvent exposure of the fluorophore.

Tyr emission splitting may be facilitated by the solvent but also by amino acid side chains with proton acceptor properties such as aspartate or glutamate^56^. In the latter case, the effect would be unaltered upon change of the buffer. To test this conjecture, experiments have been conducted in a Tris-HCl buffer. Data of Supplementary Fig. 8 show that the Tyr emission splitting is not unique to the phosphate, but the effect is stronger in phosphate than in Tris buffer (see more detail in the legend of Supplementary Fig. 8). Conceivably, Tris can weaken the OH bond of Tyr side chain by H-bonding with both hydrogen and oxygen atoms by its hydroxyl and amino groups but to a lesser extent than HPO_4_^2-^. CD spectra of all four peptides in Tris buffer were similar to those in phosphate buffer (see Fig. 3 upper row and Supplementary Fig. 9), indicating no secondary structural differences, as expected. Splitting of Tyr emission also occurred in an unbuffered solution of 25 mM NaCl, although with a smaller red-shift effect (Supplementary Fig. 10). This is exemplified for AβpE_3-42_ at λ_ex_ = 230 nm: the red-shifted component was located at 338-340 nm in phosphate and Tris buffers and at 327-336 nm in the unbuffered solution (cf. green lines in Fig. 3h, Supplementary Figs. 8e and 10). Also, the intensity of the red-shifted component for the extruded peptide was much stronger in phosphate buffer than in Tris buffer and in the unbuffered solution. While the involvement of amino acids with proton acceptor groups cannot be ruled out, clear differences between phosphate and Tris buffers and the unbuffered solution suggest that the splitting of Tyr fluorescence is caused by the solvent and is stronger when strong H-bonding acceptors and donors are present in the buffer.

The structural features of the peptides have been assessed in additional experiments at 37 °C to ensure those features are maintained at a physiological temperature. Both CD and fluorescence data showed that the peptides’ secondary structure and Tyr fluorescence splitting features at 37 °C were similar to those seen at 20 °C. Details are presented in the legends to Supplementary Figs. 11 and 12.

Overall, fluorescence and CD data indicate that Aβ_1-42_ forms tightly packed β-sheet structure and in membranes acquires a fraction of α-helix. Aβ_1-40_ forms less stable and more solvent-exposed β-sheet structure, and the vesicles induce partially unordered structure without protecting from solvent, indicating its poor ability to insert into the membranes. AβpE_3-42_ and AβpE_3-40_ form β-sheet structure in buffer and solvent-protected α/β structure in membranes, indicating membrane insertion. Thus, a correlation is established between the β-sheet propensity, membrane insertion, and channel forming capabilities of the four Aβ peptides.

### Structure and orientation of the peptides in membranes from polarized ATR-FTIR spectroscopy

**Fig. 4:**
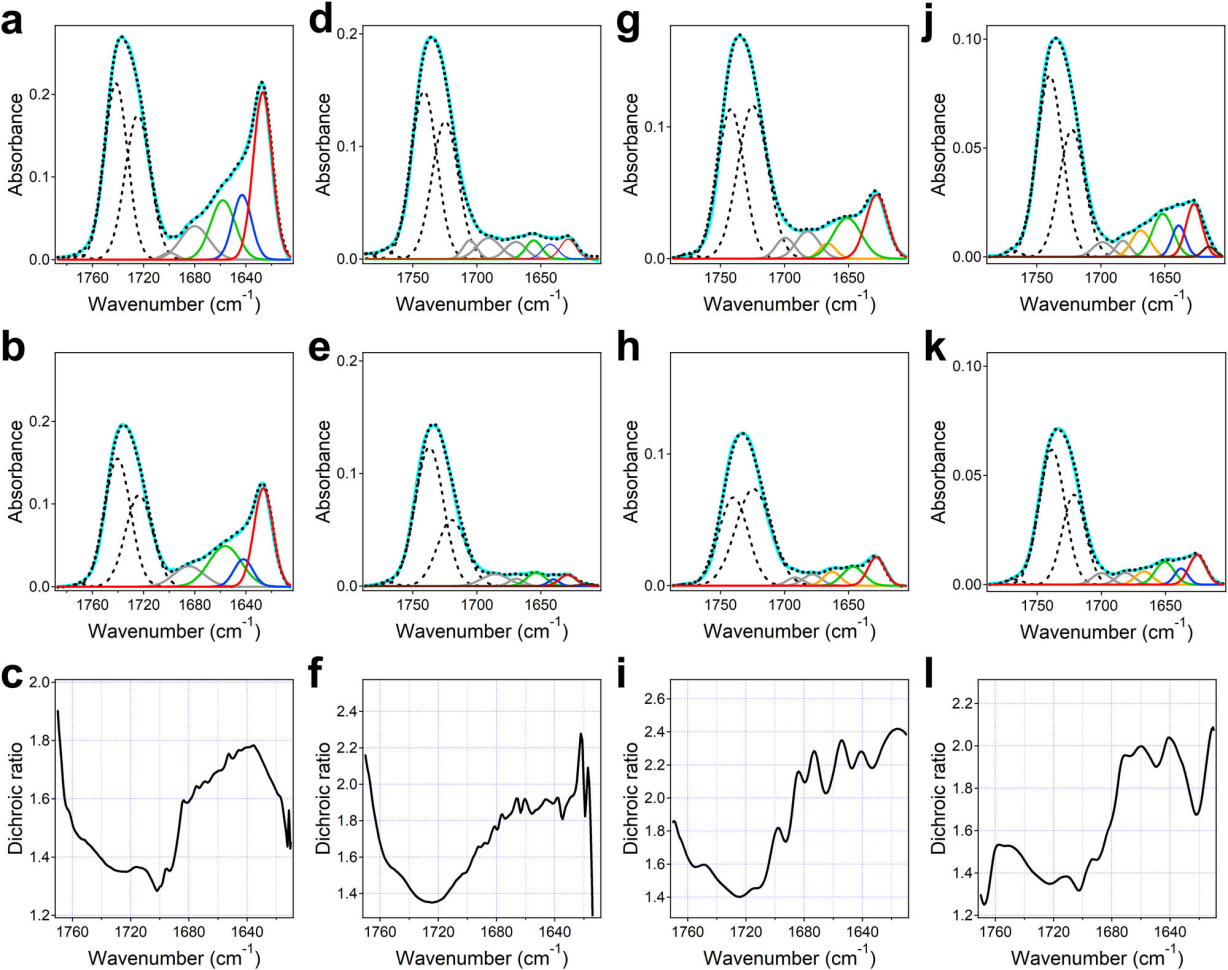
ATR-FTIR spectra of the peptides. Aβ_1-42_ (**a**, **b**), Aβ_1-40_ (**d**, **e**), AβpE_3-42_ (**g**, **h**) and AβpE_3-40_ (**j**, **k**) reconstituted in lipid multilayers composed of 60 mol % POPC, 30 mol % POPG, and 10 mol % cholesterol at 1:50 peptide-to-total lipid molar ratio, hydrated by a D_2_O-based buffer (25 mM NaCl, 25 mM Na,K-phosphate, pD 7.2), at parallel (first row) and perpendicular (second row) polarizations of the incident light relative to the plane of incidence. The measured spectrum is shown in solid line colored cyan, and the fitted curve is shown as black dotted line. Spectral components in lipid carbonyl and peptide amide I regions are presented as follows: lipid carbonyl components: black dashed lines; turn structures: gray; α_II_-helix: orange; α-helix: green; unordered: blue; β-sheet: red; side chains: brown. Panels (**c**–**f**–**i**–**l**) show the dichroic spectra, i.e., the ratio of spectra at II and ⊥ polarizations, for Aβ_1-42_, Aβ_1-40_, AβpE_3-42_, and AβpE_3-40_, respectively. Three independent experiments have been conducted with similar results.

Polarized attenuate total reflection Fourier transform infrared (ATR-FTIR) spectroscopy was used to gain information on the secondary structure of the peptides reconstituted in lipid multilayers and the orientation relative to the membrane. Figure 4 shows the ATR-FTIR spectra of all four peptides at parallel and perpendicular polarizations of the incident light with respect to the plane of incidence along with the spectral components, as well as the dichroic spectra, in the lipid carbonyl stretching and the peptides’ amide I regions. The lipid carbonyl group stretching vibration generates an absorbance band with two components at 1742 cm^−1^ and 1728 cm^−1^, which correspond to dehydrated and hydrated C = O groups, respectively; H-bonding with water weakens the C = O covalent bond and thereby decreases the vibrational frequency^57^.

Significant differences have been detected between the secondary structures of the peptides in supported membranes. Aβ_1-42_ displayed ~46% β-sheet and ~19% α-helix structures, the rest of the peptide being in turn, irregular, or other conformations (Table 1). In contrast, Aβ_1-40_ mostly adopted turn, irregular, or other conformations (68%) with only ~17% β-sheet and ~15% α-helix. The pyroglutamylated peptides had 27–35% β-sheet and 21–25% α-helix. In reality, a fraction of turns is located between β-strands and should be counted as part of the β-sheet structure. The pyroglutamylated peptides displayed an additional amide I component in the 1666-1663 cm^−1^ region, which has been assigned to α_II_-helix, i.e. a helical structure with tilted amide plane and with weaker helical H-bonding^58^. This α_II_-helical structure constituted around 9–12% (3.5–4.6 amino acid residues), i.e. about one helical turn likely located at the edge of the regular α-helix. Given the spectral overlap of type II β-turn and α_II_-helical structures^59^, these ~4 amino acid residues may alternatively be assigned to β-turn structure. These results are consistent with CD data (Fig. 3a, d, g, j and Supplementary Figs. 9, 11), indicating an ordered, mostly β-sheet structure of Aβ_1-42_, mostly unordered (or turn) conformation of Aβ_1-40_, and α/β structure of AβpE_3-42_ and AβpE_3-42_ in lipid membranes.

**Table 1:** Amide I wavenumbers (ν), fractions (*f*), and numbers of amino acid residues (*N*) for α-helix, β-sheet, unordered (ρ) structures, and turn and “other” structures (other), derived from ATR-FTIR spectra

	Aβ_1-42_	Aβ_1-40_	AβpE_3-42_	AβpE_3-40_
ν_α_(cm^−1^)	1659–1657^a^	1656–1654	1651–1646 1666–1663^b^	1652-1651 1668-1666^b^
*f*_α_	0.194 ± 0.0236	0.153 ± 0.0170	0.254 ± 0.0548 0.088 ± 0.0401^b^	0.212 ± 0.0396 0.121 ± 0.0421^b^
*N*_α_	8.15 ± 0.992	6.12 ± 0.680	10.16 ± 2.193 3.52 ± 1.606^b^	8.06 ± 1.504 4.60 ± 1.600^b^
ν_β_(cm^−1^)	1627	1629	1628	1627–1625
*f*_β_	0.459 ± 0.0367	0.170 ± 0.0190	0.351 ± 0.0521	0.270 ± 0.0419
*N*_β_	19.27 ± 1.541	6.80 ± 0.760	14.04 ± 2.0846	10.27 ± 1.591
ν_ρ_(cm^−1^)	1643–1642	1642–1640	1641–1637	1639–1637
*f*_ρ_	0.188 ± 0.0150	0.140 ± 0.0525	0.065 ± 0.0596	0.176 ± 0.0809
*N*_ρ_	7.90 ± 0.626	5.60 ± 2.100	2.60 ± 2.383	6.70 ± 3.0747
ν_other_(cm^−1^)	1700–1678	1705-1669	1699-1678	1700-1681
*f*_other_	0.159 ± 0.0151	0.537 ± 0.0551	0.242 ± 0.0549	0.221 ± 0.0405
*N*_other_	6.68 ± 0.632	21.48 ± 2.204	9.68 ± 2.197	8.37 ± 1.539

The range of wavenumbers and mean  ±  standard deviation values have been determined from three independent experiments.

^a^Wavenumbers of ⊥ spectra are usually slightly lower than those of ǁ spectra.

^b^These components have been assigned to α_II_-helix, although β-turn structure is also possible.

Notably, the amide I intensity of Aβ_1-40_ relative to that of the lipid C = O band is much weaker compared to Aβ_1-42_ (Fig. 4a, b vs. d, e), indicating that a significant part of Aβ_1-40_ is washed away during injection of the buffer owing to its more hydrophilic nature. AβpE_3-42_ and AβpE_3-40_ have intermediate amide I intensities. This is consistent with fluorescence data showing a most efficient membrane insertion and solvent protection for Aβ_1-42_ and solvent exposure for Aβ_1-40_ and the pyroglutamylated peptides (Fig. 3, Supplementary Figs. 8, 12).

The average orientation of β-strands of membrane-embedded peptides with respect to the membrane normal was deduced from polarized ATR-FTIR studies and varied between 〈β〉 = 30 and 40 degrees (Supplementary Table 1), similar to the respective angle of mitochondrial and bacterial porins, which form β-barrel structure^60^. The α-helices were tilted obliquely at 50-65 degrees relative to the membrane normal.

### Effect of Aβ peptides on lipid membranes

The lipid acyl chain order parameter was determined from polarized ATR-FTIR spectra in the methylene (CH_2_) stretching region to (a) ensure a meaningful structural order of membrane lipids and (b) assess the possible effects of the peptides on membrane structure. The CH_2_ groups undergo asymmetric and symmetric stretching vibrations that generate absorbance bands around 2920 cm^−1^ and 2850 cm^−1^, respectively^61^ (Supplementary Fig. 13). As seen from Supplementary Table 2, the order parameter of plain lipid was ~0.44, indicating well organized membranes (see legend to Supplementary Fig. 13 for justification). The presence of peptides in the membranes exerted nontrivial effects on the lipid order. Aβ_1-42_ increased the lipid order whereas the other three peptides decreased *S*_L_ (Supplementary Table 2). The difference between the effect of Aβ_1-42_ and the other three peptides was statistically significant as judged from the *p-*values when comparing *S*_L_ in the presence of Aβ_1-42_ with those in the presence of Aβ_1-40_, AβpE_3-42_, and AβpE_3-40_ (*p* = 0.0468, 0.0374, and 0.0089, respectively). This finding suggests a unique property of Aβ_1-42_ compared to the other peptides, which may be related to its ability to form stable ion-conducting channels in membranes. Aβ_1-40_ and AβpE_3-40_, on the other hand, decreased the lipid order, an effect possibly related to a membrane destabilization mechanism of ion conductance induced by these peptides.

### Morphology of Aβ peptides in lipid membranes from AFM

The morphological features of the four Aβ peptides without and with reconstitution in lipid membranes were probed by AFM. First, peptides freshly suspended in aqueous buffer and incubated under fibrillization conditions (24 h, 37 °C) were studied. All freshly suspended peptides showed a distribution of monomers and oligomers in the height range of 1–5 nm (Supplementary Fig. 14). Aβ_1-42_ and Aβ_1-40_ showed a uniform distribution of monomers and oligomers in the height range 1–3 nm (section profile below Supplementary Fig. 14a, b) while AβpE_3-42_ and AβpE_3-40_ displayed higher ordered oligomeric structures with heights ranging from 2 nm to 5 nm above the mica plane (section profile below Supplementary Fig. 14c, d).

Under fibrillogenesis conditions, Aβ_1-42_ and Aβ_1-40_ showed highly dense, entangled fibrous structures (Supplementary Fig. 14e, f). In contrast, the pyroglutamylated peptides showed a very sparse distribution of individual fibrils without entanglement (Supplementary Fig. 14g, h). Even though all peptides showed a positive thioflavin-T signal, the fibril distribution for the pyroglutamylated variants was not easily visualized as compared to the unmodified peptides and some higher order, large-sized oligomers could be observed, which may indicate incomplete fibril elongation.

**Fig. 5:**
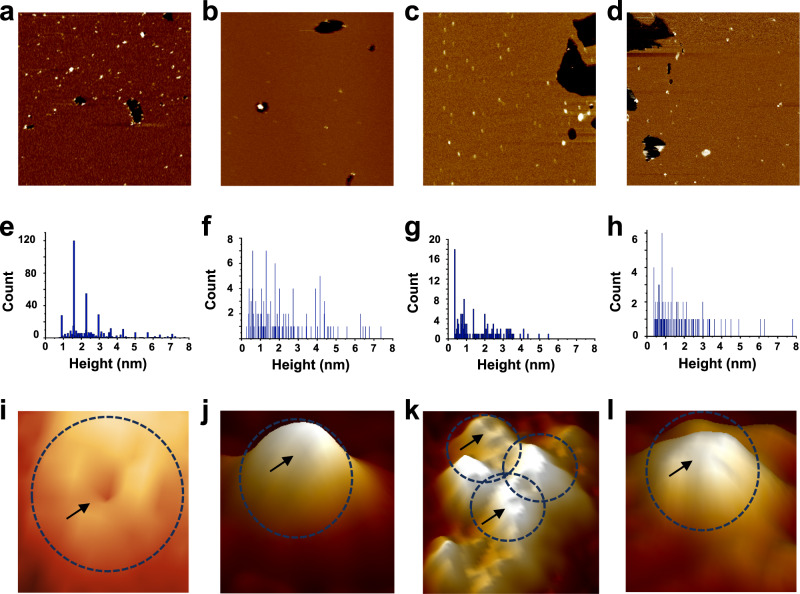
AFM height images of the peptides reconstituted in POPC:POPG:cholesterol (6:3:1 molar ratio) membranes at 1:500 peptide:lipid molar ratio. (**a**) Aβ_1-42_ (scan size = 1.6 μm × 1.6 μm), (**b**) Aβ_1-40_ (scan size = 1.5 μm × 1.5 μm), (**c**) AβpE_3-42_ (scan size = 2.0 μm × 2.0 μm), (**d**) AβpE_3-40_ (scan size = 1.7 μm × 1.7 μm). For the upper-row images, the darkest part of the image corresponds to the mica surface, and the z-scale is 0–5 nm. The center row presents histograms of particle height above the bilayer plane for Aβ_1-42_ (**e**) Aβ_1-40_ (**f**) AβpE_3-42_ (**g**) AβpE_3-40_ (**h**) combined from 5 separate images per peptide. The lower row presents close-up 3D views of ion channel/pore-like structures formed by Aβ_1-42_ (panel (**i**) scan size = 20 nm × 20 nm), Aβ_1-40_ (panel (**j**) scan size = 30 nm × 30 nm), AβpE_3-42_ (panel (**k**) scan size = 30 nm × 30 nm), and AβpE_3-40_ (panel (**l**) scan sizes = 20 nm × 20 nm). For all high-resolution images (lower row), the z-scale is 0–2 nm. The dashed circles represent structures resembling ion channels and the arrows indicate the position of the pore. The buffer was 300 mM KCl and 10 mM HEPES, pH 7.4. Three independent experiments have been conducted with similar results.

Next, the morphological features of the peptides reconstituted in lipid membranes were examined (Fig. 5). In all cases, the image analysis showed two populations of peptide oligomers, one protruding from the membrane 0.5–2.0 nm above the bilayer surface, assigned to membrane-inserted structures, and the other exceeding 2 nm, assigned to a membrane-adsorbed peptide pool (Fig. 5e–h).

Among the membrane inserted fractions, a small sub-population exhibited pore-like morphology. These structures were better resolved in case of Aβ_1-42_ and featured annular assemblies composed of 4–6 units with an outer diameter of ~16 nm (Fig. 5i). The other three peptides exhibited oligomeric structures in comparable height ranges, although the histogram of Aβ_1-40_ was shifted towards larger heights above membrane surface (Fig. 5f), consistent with membrane-adsorbed rather that embedded mode of membrane binding of this peptide. Pore-like morphologies were not identified for Aβ_1-40_ and AβpE_3-40_ (Fig. 5j, l). In the case of AβpE_3-40_, although an apparent subunit like topology was weakly observed (Fig. 5l), AFM tip convolution effects prevented from fully resolving the subunits. For AβpE_3-42_, annular, pore-like structures were seen with outer diameter from 9 to 15 nm (Fig. 5k). Clusters of pore-like structures were seen for this peptide as well. Overall, apparent pore-like structures were resolved more readily for Aβ_1-42_, followed by AβpE_3-42_, consistent with the patch clamp data showing regular step-like channel behavior for these two peptides.

## Discussion

In this work, membrane channel formation by the most abundant and toxic forms of Aβ peptides, i.e., Aβ_1-42_, Aβ_1-40_, AβpE_3-42_, and AβE_3-40_, have been analyzed and correlated with their structural and morphological features. The peptides have been reconstituted in lipid membranes composed of POPC, POPG, and cholesterol to mimic the fluidity, the anionic surface charge, and cholesterol content of neuronal membranes^62^. In fact, channel conductances recorded in this work range from 100 pS to 1000 pS, similar to those induced by Aβ peptides in cell membranes^25,30^. Analysis of the peptides under identical conditions allowed identification of a clear correlation between the β-sheet content, degree of membrane insertion, morphological features, and channel formation abilities of the peptides.

Aβ_1-42_ and Aβ_1-40_, which differ by only two amino acids (Ile_41_Ala_42_), exhibit strikingly diverse structural and channel forming properties. Aβ_1-42_ contains the largest β-sheet fraction (2.7-fold more than Aβ_1-40_), efficiently inserts into lipid membranes, forms annular supramolecular assemblies, and produces single-channel-like currents resembling those observed in cell membranes^22,30^. Aβ_1-40_, on the other hand, displays a conformation rich in turn and unordered structures, with little β-sheet, fails to effectively embed in membranes, shows irregular morphology, and produces infrequent bursts of current, again similar to those detected in neuronal membranes^25^. These data lead to a conclusion that Ile_41_ and Ala_42_ play a crucial role in promoting β-sheet formation and membrane insertion of the Aβ peptides and subsequent channel formation. This is consistent with effective ion channel formation in membranes of HEK293 cells by Aβ_1-42_ but not Aβ_1-40_, which was attributed to differences in membrane interaction and insertion abilities of the two Aβ species^30^. Given the conflicting data reported for membrane pore formation by Aβ_1-40_ (see Introduction), our results argue in favor of perturbation of cellular or lipid membranes by this peptide via a mechanism different from regular channels that switch between open and closed states. This work identifies another distinction between Aβ_1-42_ and Aβ_1-40_: Aβ_1-42_ increases whereas Aβ_1-40_ decreases the structural order of lipid acyl chains (Supplementary Table 2). Conceivably, the regular β-barrel-like channel structures formed by Aβ_1-42_ stabilizes the structure of surrounding lipids while Aβ_1-40_ exerts an opposite, membrane destabilizing effect, which possibly contributes to induction of irregular current spikes.

The pyroglutamylated peptides show a combination of stepwise and burst-like current patterns. Notably, these peptides are able to form pores of larger conductance compared to their unmodified counterparts. An earlier study showed that 74% and 21% of AβpE_3-42_ formed channels in lipid bilayers with conductance < 100 pS and between 100 pS and 200 pS, respectively, while for Aβ_1-42_ these percentages were 91% and 5%, indicating overall larger channels formed by AβpE_3-42_^39^. Consistent with this, the conductance of L_o2_ state of AβpE_3-42_, observed in this work, is 300 pS, much larger than the 100 pS channels formed by Aβ_1-42_. Channel data for AβpE_3-40_ are not available, but our data indicate this peptide is able to form even larger channels, with conductance reaching 1 nS. It is interesting to note in this context that AβpE_3-40_ exerted stronger cytotoxic effect on cultured rat hippocampal neurons and cortical astrocytes than Aβ_1-40_, Aβ_1-42_, or AβpE_3-42_^63^, which might be related to its ability to form larger channels in cell membranes. The high frequency of opening of relatively large channels formed by AβpE_3-42_ and AβpE_3-40_ is likely to contribute to their augmented cytotoxicity, partially confirmed by induction of more efficient Ca^2+^ influx into neurons caused by AβpE_3-42_ as compared to Aβ_1-42_^7^.

Channel internal diameters (*d*) were estimated using the measured single channel conductance values and the structural data, as described in the Supplementary Methods and presented in the Supplementary Table 3. For each peptide, a specific channel length (*l*) has been determined based on data of Table 1 and Supplementary Table 1. As the best single channel forming peptides, such as Aβ_1-42_, contained large fractions of β-sheet, with the β-strands tilted from the membrane normal at an angle of 30-40 degrees, these simulations have been conducted assuming a β-barrel-like structure for the peptides. Two values for the resistivity of the salt solution were used, the bulk value and the 5-fold elevated “corrected” value, which we consider more realistic as justified in the Supplementary Methods. The channel diameters, using the corrected solution resistivities, were around 0.56 nm for Aβ_1-42_ channels with 100 pS conductance and around 0.57 nm and 0.83 nm for AβpE_3-42_ channels with 150 pS and 300 pS conductance (Supplementary Table 3). For AβpE_3-40_ channel conductances of 200 pS, 400 pS, 640 pS, and 1040 pS, we obtained *d* = 0.59 nm, 0.86 nm, 1.12 nm, and 1.48 nm, respectively. Using the bulk resistivity value resulted in nearly 2-fold narrower pore diameters. Aβ_1-40_ showed a minimal β-sheet fraction, corresponding to ~7 amino acid residues (Table 1), which, when coupled with the strand tilt angle of ~35 °, can make a 2 nm long barrel-like structure, about half of the membrane thickness. Based on these results, Aβ_1-42_ is likely to form stable transmembrane channels, consistent with clear step-like current levels and increased lipid order (Supplementary Table 2). AβpE_3-42_ may form transmembrane channels as well (*l* = 3.84 nm) with minimal effect on the lipid order, whereas AβpE_3-40_ (*l* ≈ 3 nm) is likely to be incompletely membrane inserted, exerting a lipid destabilizing effect (Supplementary Table 2). Aβ_1-40_ (*l* = 2 nm) is not likely to form a transmembrane channel, consistent with the unique spiky current features generated by this peptide (Figs. 1, 2). Instead, it may cause membrane permeabilization by the “carpet” or detergent-like mechanisms. Still, Aβ_1-40_ is included in Supplementary Table 3 to show that if it were to form a β-barrel, then its pore diameter would vary between 0.34 and 0.66 nm, using the corrected solution resistivity.

Internal channel diameters for Aβ_1-42_ in lipid bilayers have been evaluated based on electrophysiological recordings, using channel lengths from 3 nm^37^ to 6 nm^30,40^, and varied between 0.7 nm and 2.1 nm^37,40^ whereas in cell membranes they varied between 1.7 nm and 2.4 nm assuming *l* = 7 nm^30^. MD simulations of Aβ_1-42_ and AβpE_3-42_ octadecamers yielded pore diameters of up to 2.2 nm (see references in ref. ^40^). Channel diameters shown in the Supplementary Table 3 are close to those reported for lipid bilayers but are smaller when compared to Aβ_1-42_ channels in cell plasma membranes^30^. This raises the possibility that cellular components, such as gangliosides or accessory proteins such as the cellular prion protein^32^, may facilitate formation of larger Aβ pores.

Interestingly, the Tyr_10_ of Aβ_1-42_ was solvent-inaccessible in the absence and presence of lipid membranes, suggesting that compact secondary and tertiary structures form in the aqueous phase and then the peptide inserts into the membrane and forms regular ion channels. The other three peptides had solvent-exposed Tyr_10_; lipid membranes either failed to protect it from the solvent (Aβ_1-40_) or shielded it partially (AβpE_3-42_) or totally (AβpE_3-40_), indicating distinct modes of membrane binding/insertion of various Aβ species. Among the four peptides studied here, only Aβ_1-42_, which is the full-length peptide in terms of possessing both the Asp_1_Ala_2_ and Ile_41_Ala_42_ residues, forms large fraction of β-sheet, effectively inserts into the membrane, displays well-defined pore like morphology, and forms regular ion channels. This is reminiscent of the inability of a N- and C-terminally truncated peptide, Aβ_4-34_, to form pore-like structures and stable pores in lipid membranes^64^. The other three peptides that lack two amino acids at one or both termini have smaller β-sheet content, are more solvent exposed, and induce irregular current bursts, albeit of large conductivity. Thus, our data uncover a delicate relationship between all four structural levels of Aβ peptides and their membrane pore formation capabilities and hence neurotoxic potentials. The presence of the N- and C-terminal residues (primary structure), the β-sheet content (secondary structure), the compactness of the tertiary fold including the N-terminal segment harboring Tyr_10_, and the supramolecular quaternary structure all are inter-related and contribute to the mode of membrane binding and pore formation. While more work needs to be done to provide more detailed insight into the molecular mechanisms of membrane pore formation by these peptides, including the specific roles of the key amino acids and high-resolution structures of Aβ membrane pores by methods such as NMR or cryo-electron microscopy or tomography^32,65^, the present data shed light on the intricate relationships between the structural propensities and membrane permeabilization features by these most important Aβ peptides.

## Methods

Information about materials used and the full protocols are available in the Supplementary Methods. Here, a brief description of all procedures is presented.

### Voltage clamp electrophysiology experiments

The lipid bilayers were prepared in a 250 μm aperture in a Derlin cuvette using POPC, POPG, and cholesterol dissolved in *n*-decane at a 6:3:1 molar ratio. The buffer used was 1 M KCl + 10 mM HEPES, pH 7.4. Bilayer formation was followed by capacitance measurements using a 90-pF minimum threshold. The voltage clamp measurements were conducted using a high-gain electrophysiology amplifier with a resistive feedback headstage (Warner Instruments, BC-535), a digitizer (Digidata 1440 A), and a workstation computer. Data were recorded with a sampling frequency of 10 kHz. An integrated 8-pole low pass Bessel filter was used to filter the data at 1.0 kHz bandwidth. For further filtering and noise reduction, the data were also collected in parallel with another Bessel filter (Warner Instruments, LPF-8) at a cutoff frequency of 60 Hz. Data analysis was carried out with Clampfit (v10.6, Axon Instruments, San Jose, CA).

Peptide samples were prepared by dissolving in 1% NH_4_OH and diluting into a working buffer (1 M KCl +10 mM HEPES, pH 7.4). The peptide was added to one of the wells of the cuvette (trans side) at a final concentration of 100 nM, incubated for 5 min, followed by current recordings at various hold voltages, i.e., −100 mV, −50 mV, 50 mV, 100 mV (the sign corresponds to the trans side). Blockade of the channels by Zn^2+^ ions was tested by adding ZnCl_2_ to the trans side.

### Electrophysiology data analysis

The current traces were analyzed in a pClamp analysis environment. Between 120 s and 180 s snippets were selected, where the electrical activity was visually clear. Amplitude histograms of these snippets were generated to display the distribution of conductance values.

Channel diameter (*d*) was estimated using the formula^30,40^:

1 $$d=\frac{\rho {g}_{{ch}}}{2}\left(1+\sqrt{1+\frac{16l}{\pi \rho {g}_{{ch}}}}\right)$$

where *ρ* is the resistivity of the salt solution inside the channel, *g*_ch_ is the measured single channel conductance, and *l* is the channel length. The length of a β-barrel-like channel was estimated based on the number of amino acid residues in the β-strand (*N*_β_), the displacement along the β-strand axis per amino acid residue (*a* = 3.48 Å), and the β-strand tilt angle (β= 30-40 degrees relative to the membrane normal): *l* = *aN*_β_cosβ. For the solution resistivity in the channel, two values have been used, i.e., the bulk valued and an empirically corrected, 5-fold higher value, as described in the Supplementary Methods file.

### AFM imaging and data analysis

For AFM imaging, the peptides were suspended in a buffer containing 300 mM KCl and 10 mM HEPES (pH 7.4) to 1 μM final concentration, vortexed for 30 s, deposited on freshly cleaved mica, incubated for ~10 min, washed 3 times with the same (imaging) buffer, and scanned. To prepare peptide/lipid samples for AFM imaging, the lipids were suspended in the imaging buffer supplemented with 3 mM CaCl_2_ and bath-sonicated to obtain unilamellar vesicles. The peptide was added at 1:1000 or 1:500 peptide-to-lipid molar ratio followed by a ~ 1 min bath-sonication. The sample was drop-casted on the freshly cleaved mica surface and incubated for 20 min at room temperature, washed 3 times with the imaging buffer and the formed supported lipid bilayers were imaged in the same buffer. AFM imaging was performed on a Multimode AFM (Bruker) controlled by a Nanoscope V controller and the images were collected in PeakForce Tapping mode with SNL-10 cantilevers (Bruker). The images were analyzed using a Nanoscope Analysis v1.5 software or the SPM analysis package Gwyddion. All images were line flattened and color scale adjusted. For generating particle height histograms, the “Particle Analysis” feature in Nanoscope Analysis was used.

### CD and fluorescence

The lyophilized lipids and peptides were dissolved in chloroform and hexafluoroisopropanol (HFIP), respectively. The peptide solution was dried by desiccation followed by addition of the buffer and vortexing for 5 min. To prepare the proteoliposomes, the chloroform solution of the lipid was combined with the HFIP solution of the peptide at a 50:1 total lipid-to-peptide molar ratio, desiccated for 1 h, suspended in the buffer and vortexed for 5 min. When needed, the peptide or proteoliposome samples were extruded through 100 nm pore-size polycarbonate membranes using an Avanti Polar Lipids mini extruder. CD and fluorescence spectra were measured using a J-810 spectropolarimeter equipped with a fluorescence attachment and a temperature controller (Jasco, Tokyo, Japan) at 20 °C and 37 °C. Ten scans were averaged for the CD spectra and 3 scans for the fluorescence spectra.

### Attenuated total reflection Fourier transform infrared spectroscopy

For ART-FTIR experiments, the chloroform solution of the lipids was mixed with the HFIP solution of the peptide at a 50:1 total lipid-to-peptide molar ratio and spread over the surface of a germanium plate (5 cm × 2 cm × 0.1 cm, cut at the 2 cm edges at a 45 ° bevel angle). The sample was dried by 1-h desiccation and assembled in a flow-through ATR sample cell (Buck Scientific, East Norwalk, CT). The buffer (25 mM NaCl + 25 mM Na,K-phosphate in D_2_O, pD 7.2) was injected into the cell to hydrate the peptide-lipid sample and FTIR spectra were recorded using a Vector-22 FTIR spectrometer (Bruker, Billerica, MA, USA) at two polarizations of the incident light, i.e., parallel and perpendicular to the plane of incidence. Transmission spectra of the blank buffer were measured separately and used as reference to obtain the respective absorbance spectra.

### ATR-FTIR data analysis

The secondary structure of the peptides was determined based on peak-fitting of the amide I FTIR spectra. First, the spectra measured at parallel and perpendicular polarizations of the incident light relative to the plane of incidence were used to obtain a “polarization-independent” spectrum, as described in the Supplementary Methods. Then, the areas of various amide I components were assigned to certain secondary structure types as follows: α-helix: 1660–1646 cm^−1^, unordered: 1645–1638 cm^−1^, β-sheet: 1637–1623 cm^−1^, turn or other structures: 1705–1661 cm^−1^. The areas of the components, corrected using the respective integrated molar absorptivities, relative to the total amide I area represented the fractions of various secondary structures.

The orientations of α-helical and β-sheet components were determined based on the dichroic ratios, *R* = *a*_II_/*a*_⊥_, where *a*_II_ and *a*_⊥_ are the amide I areas of a given structural component at parallel and perpendicular polarizations of the incident light, respectively. The order parameter of lipid acyl chains in supported membranes was determined based on the dichroic ratio of the methylene stretching vibrations in the spectral region 3015 cm^−1^–2810 cm^−1^.

### Reporting summary

Further information on research design is available in the Nature Portfolio Reporting Summary linked to this article.

### Supplementary information

Supplementary Information

Peer review file

Reporting Summary

## Data Availability

Electrophysiological data for all four peptides at various applied voltages and respective conductance histograms, the effect of Zn^2+^ ions on ion-conducting channels, light scattering, additional fluorescence, CD, and FTIR spectra as well as AFM images are provided in the Supplementary Information file. Any other data such as CD spectra of unextruded samples in the presence of lipid or thioflavin-T spectra, can be obtained from the authors upon request.
