# Supplementary material for: The structure of tyrosine-10 favors ionic conductance of Alzheimer’s disease-associated full-length amyloid-β channels
Source: Nat Commun. 2024 Feb 13;15:1296. doi: 10.1038/s41467-023-43821-y (PMC10864385; doi:10.1038/s41467-023-43821-y)
Supplement: Supplementary file 1 — Supplementary Information [file 41467_2023_43821_MOESM1_ESM.pdf]

Supplementary Information

The structure of tyrosine-10 favors ionic conductance of Alzheimer's disease-associated full-length amyloid-β channels

Abhijith G. Karkisaval[1], Rowan Hassan[2], Andrew Nguyen[3], Benjamin Balster[3], Faisal Abedin[2,4], Ratnesh Lal[1,3,*] & Suren A. Tatulian[2,*]

[1] Department of Mechanical and Aerospace Engineering, University of California San Diego, La Jolla, CA

[2] Department of Physics, University of Central Florida, Orlando, FL

[3] Department of Bioengineering, University of California San Diego, La Jolla, CA

[4] Currently at the Department of Biology, Xavier University of Louisiana, New Orleans, LA

* Correspondence and requests for materials should be addressed to R.L. (rlal@ucsd.edu) or S.A.T. (statulia@ucf.edu)

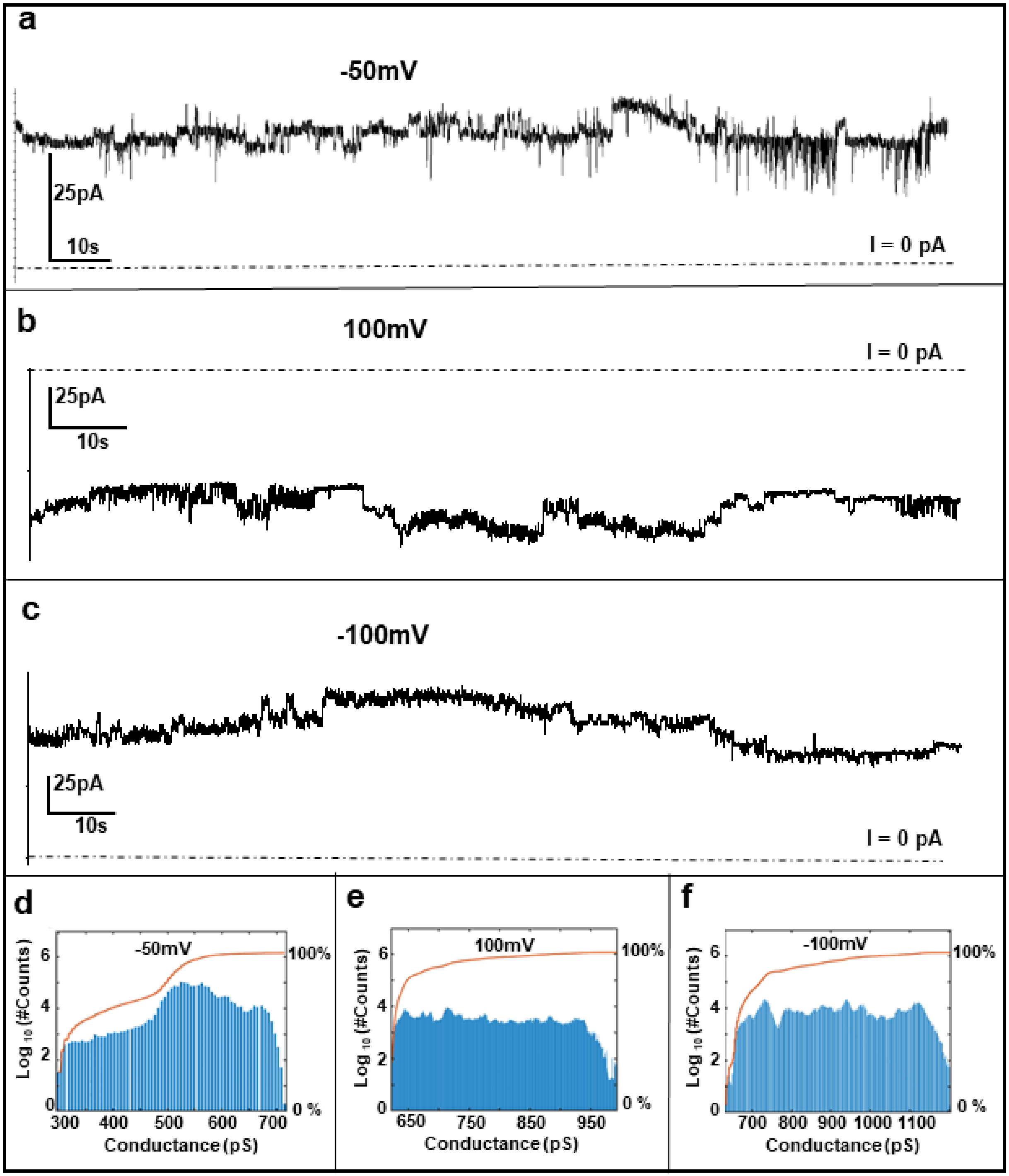

Supplementary Figure 1. Electrophysiological recordings (**a**, **b**, **c**) and histograms (**d**, **e**, **f**) of Aβ_{1-42} in lipid bilayers composed of 60 mol % 1-palmitoyl-2-oleoyl-phosphatidylcholine (POPC), 30 mol % 1-palmitoyl-2-oleoyl-phosphatidyglycerol (POPG), and 10 mol % cholesterol at hold voltages of -50 mV, +100 mV and -100 mV, as indicated. The buffer was 1 M KCl +10 mM HEPES, pH 7.4. In histograms, the total counts/bin are expressed in log scale in the left y-axis (blue bars) and the cumulative counts (0-100%) per conductance level are indicated in the right y-axis (red line). At least five independent experiments have been conducted with similar results.

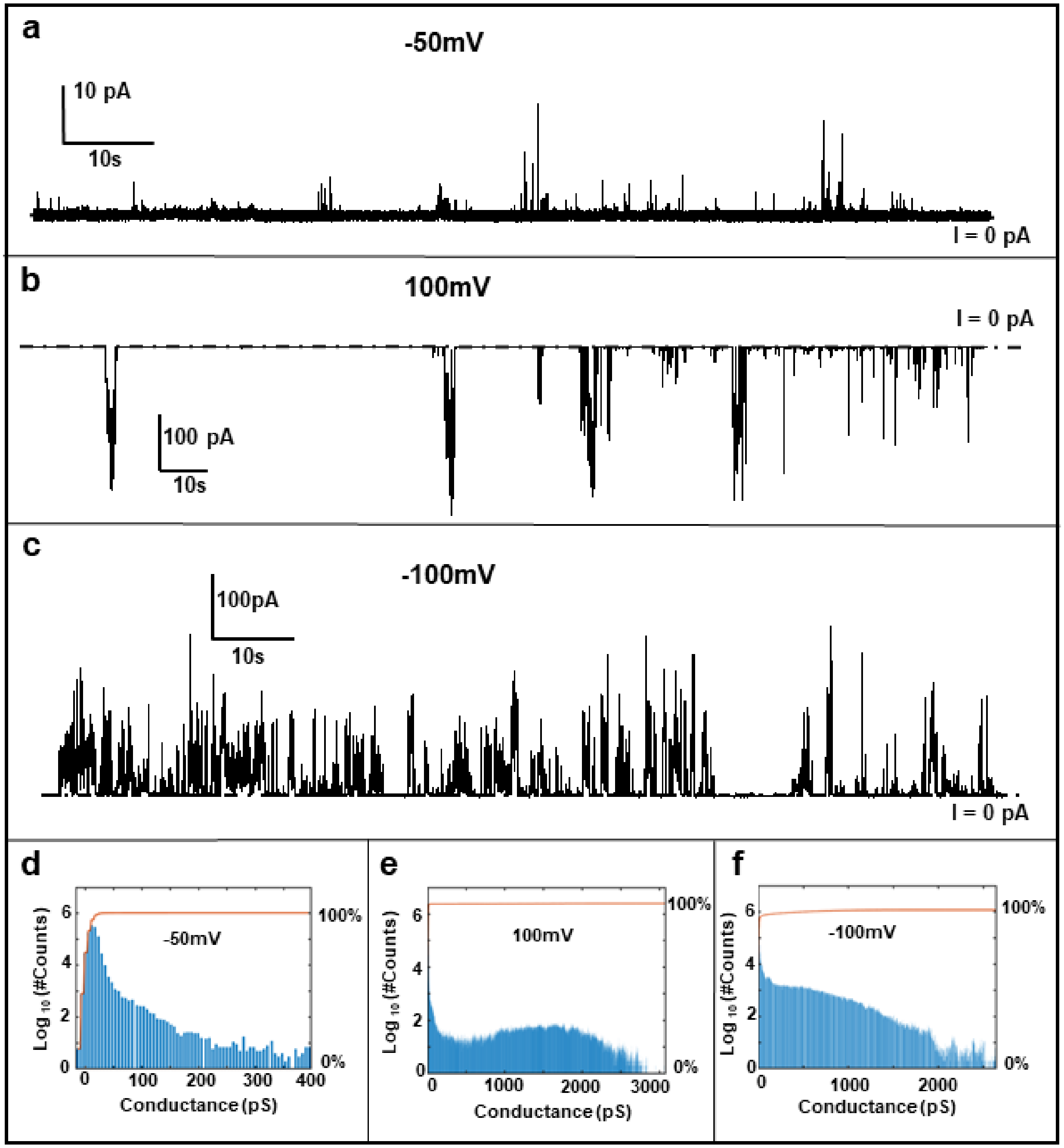

Supplementary Figure 2. Electrophysiological recordings (**a**, **b**, **c**) and histograms (**d**, **e**, **f**) of Aβ1-40 in lipid bilayers (POPC:POPG:cholesterol = 6:3:1 molar ratio) at hold voltages of -50 mV, +100 mV and -100 mV, as indicated. The buffer was 1 M KCl + 10 mM HEPES, pH 7.4. In histograms, the total counts/bin are expressed in log scale in the left y-axis (blue bars) and the cumulative counts (0-100%) per conductance level are indicated in the right y-axis (red line). At least five independent experiments have been conducted with similar results.

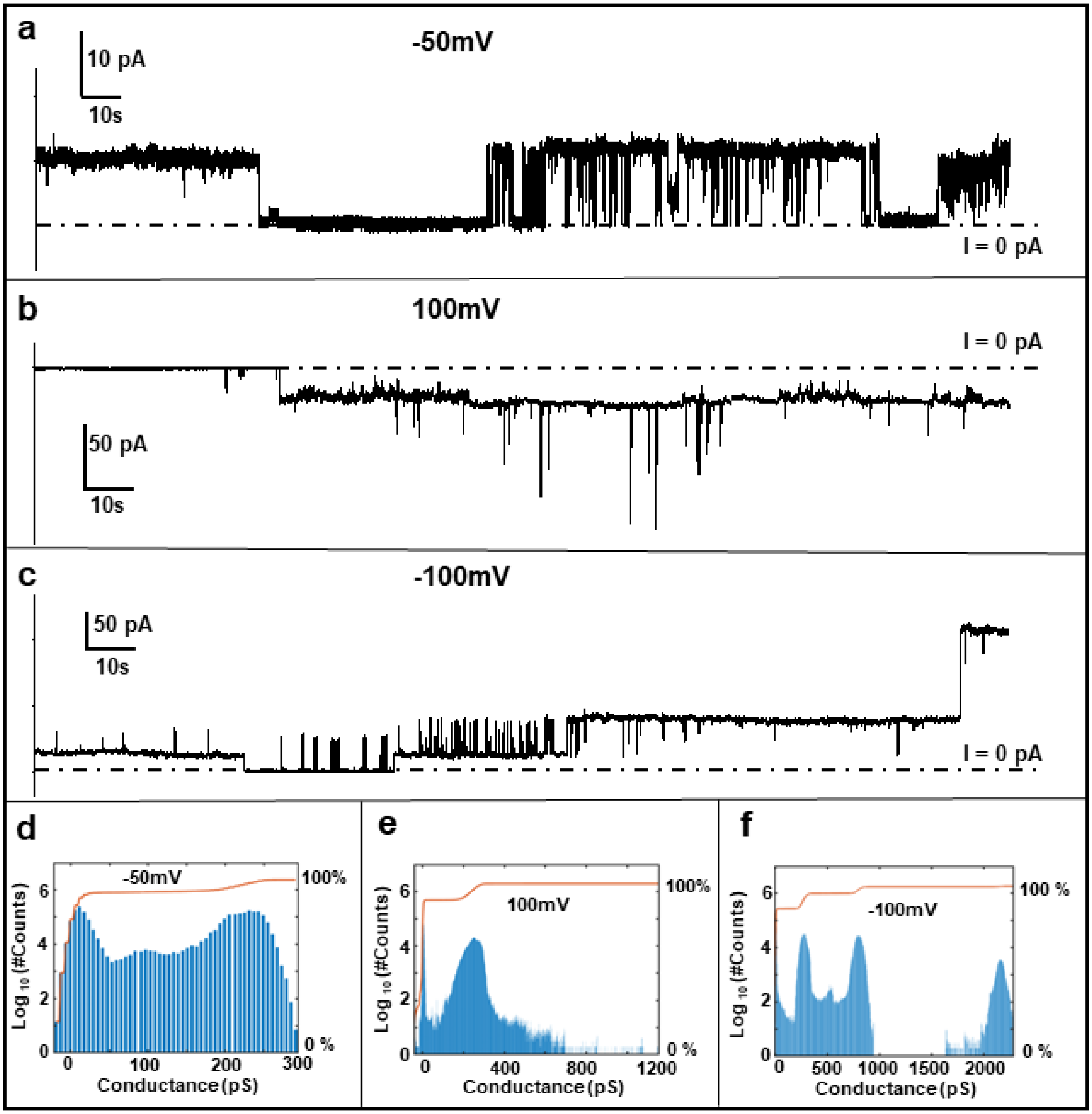

Supplementary Figure 3. Electrophysiological recordings (**a**, **b**, **c**) and histograms (**d**, **e**, **f**) of AβpE$_{3-42}$ in lipid bilayers (POPC:POPG:cholesterol = 6:3:1 molar ratio) at hold voltages of -50 mV, +100 mV and -100 mV, as indicated. The buffer was 1 M KCl +10 mM HEPES, pH 7.4. In histograms, the total counts/bin are expressed in log scale in the left y-axis (blue bars) and the cumulative counts (0-100%) per conductance level are indicated in the right y-axis (red line). At least five independent experiments have been conducted with similar results.

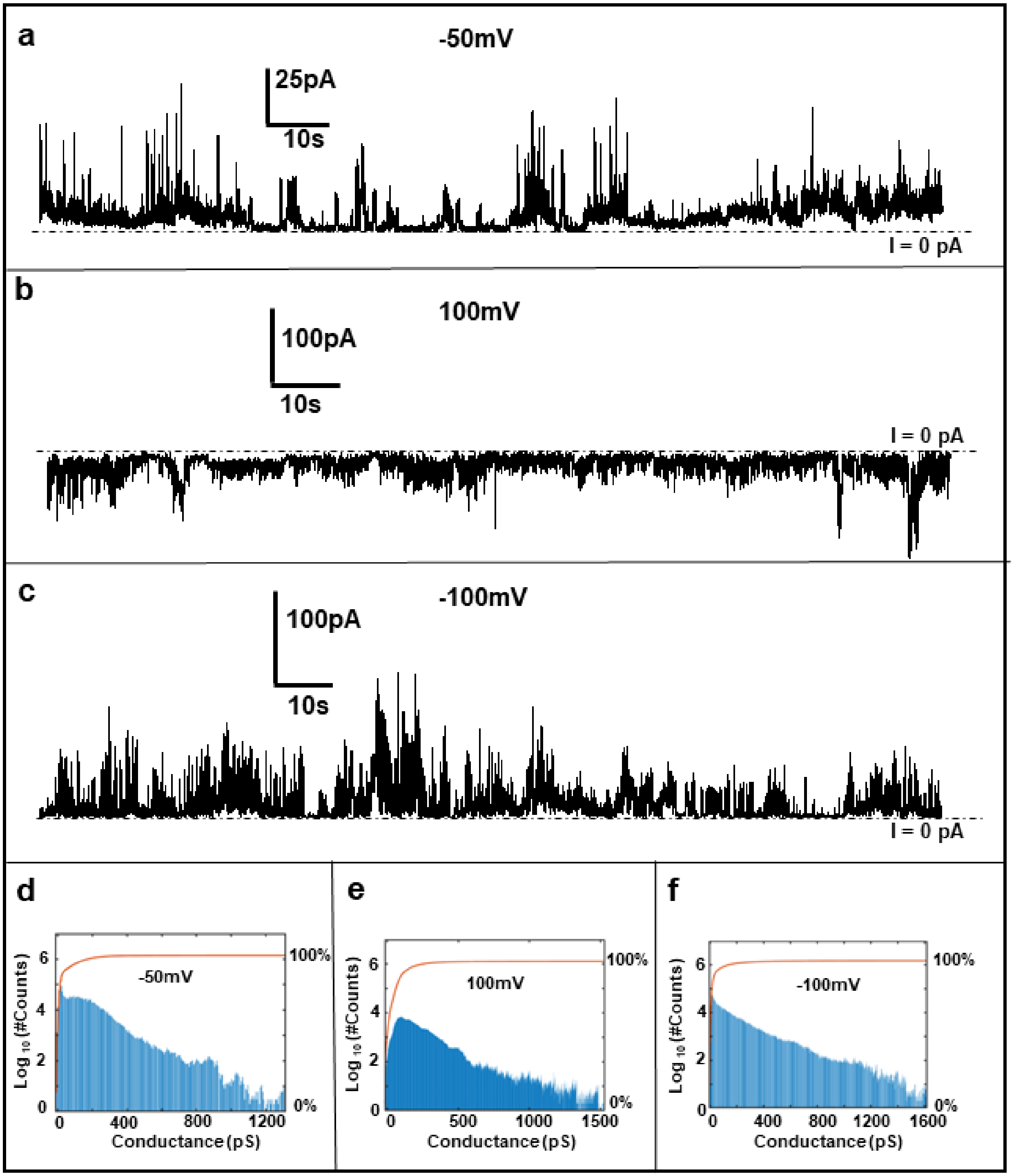

Supplementary Figure 4. Electrophysiological recordings (**a**, **b**, **c**) and histograms (**d**, **e**, **f**) of AβpE$_{3\text{-}40}$ in lipid bilayers (POPC:POPG:cholesterol = 6:3:1 molar ratio) at hold voltages of -50 mV, +100 mV and -100 mV, as indicated. The buffer was 1 M KCl +10 mM HEPES, pH 7.4. In histograms, the total counts/bin are expressed in log scale in the left y-axis (blue bars) and the cumulative counts (0-100%) per conductance level are indicated in the right y-axis (red line). At least five independent experiments have been conducted with similar results.

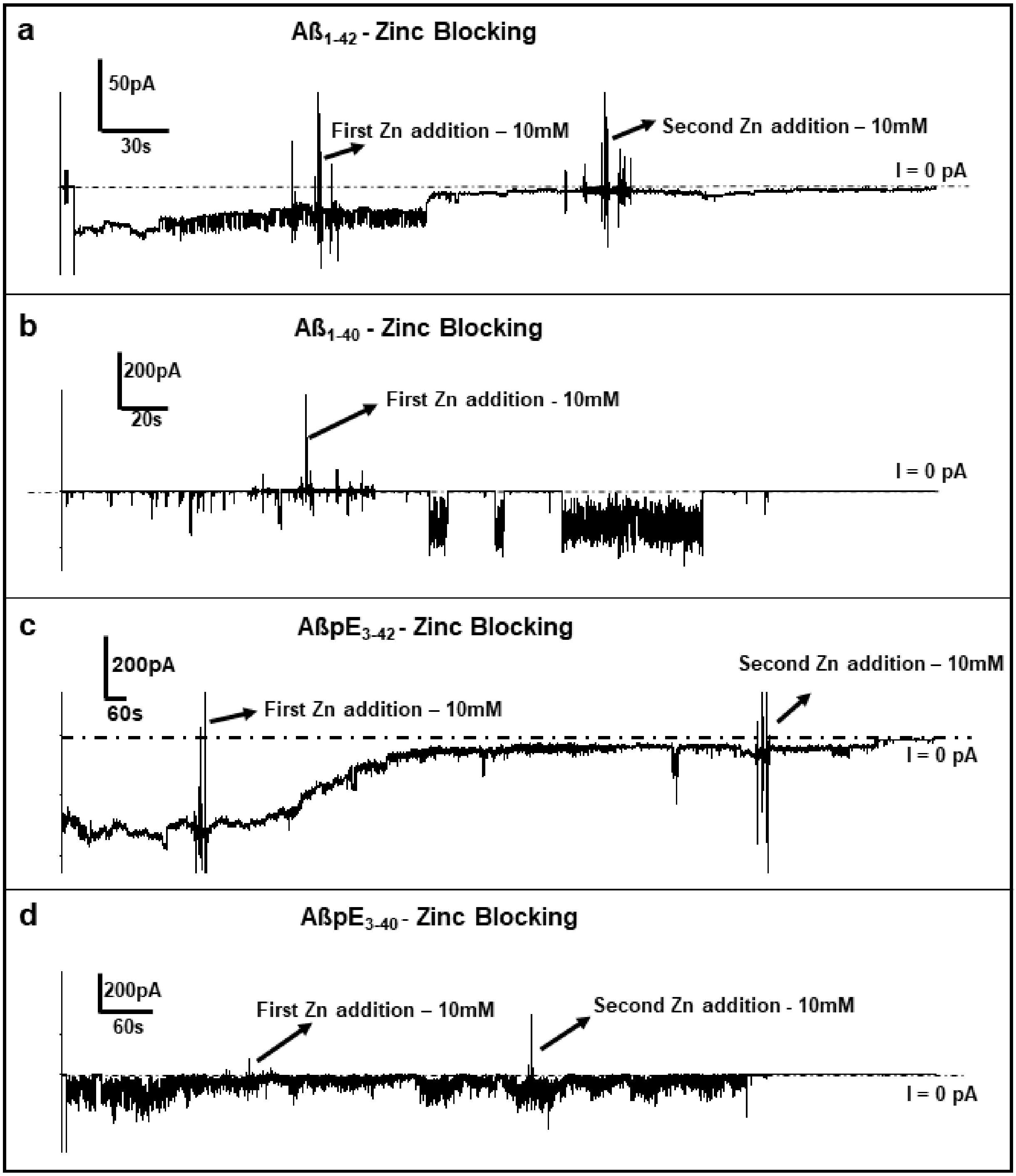

Supplementary Figure 5. Ion channel blocking by Zn^{2+} ions for Aβ1-42 (**a**), Aβ1-40 (**b**), AβpE3-42 (**c**), and AβpE3-40 (**d**). Electrophysiological traces of the peptides at +100 mV hold potential, with 10 mM $ZnCl_2$ added to the trans chamber. In all cases except for Aβ1-40, $ZnCl_2$ was added twice, marked by arrows. The buffer was 1 M KCl +10 mM HEPES, pH 7.4. At least five independent experiments have been conducted with similar results.

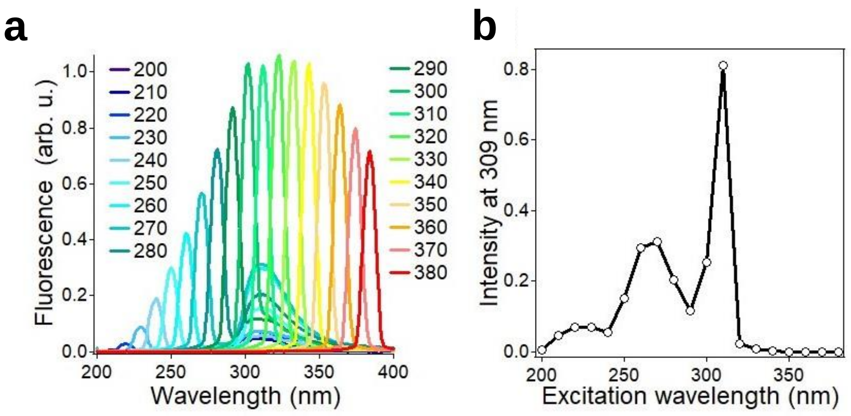

Supplementary Figure 6. (**a**): Fluorescence spectra of A$\beta_{1\text{-}42}$ in buffer (25 mM NaCl + 25 mM Na,K-phosphate, pH 7.2) with incident light of 200 – 380 nm, as indicated. (**b**): Emission intensity at 309 nm as a function of the incident light wavelength. Temperature was 20°C. Three independent experiments have been conducted with similar results. These spectroscopic features reflect the fact that the total extinction of the incident light is due to absorption and scattering, and that in a certain spectral region, absorption results in fluorescence. Although there is only one Tyr (position 10) and three Phe residues (positions 4, 19, 20) in the peptide, the fluorescence is dominated by Tyr due to its larger quantum yield (1). The spectra display Rayleigh scattering at each excitation wavelength (λ_{ex}), as well as Tyr fluorescence around 309 nm when $\lambda_{ex} \leq 290$ nm. Data of panel **b** show two regions of λ_{ex} that produce maximum Tyr fluorescence, i.e., 220-230 nm and 260-280 nm, which are the $\pi\pi^*$ transitions from the ground stat to the second and first excited singlet states, i.e., $S_0 \rightarrow S_2$ and $S_0 \rightarrow S_1$, respectively (1, 2). (The peak around 310 nm is due to scattered light, not fluorescence.) In the absence of absorption, i.e. above 300 nm, Rayleigh scattering is proportional to λ^{-4} and decreases with increasing λ whereas below 300 nm absorption dominates and consequently scattering decreases with decreasing λ, as seen in panel **a**.

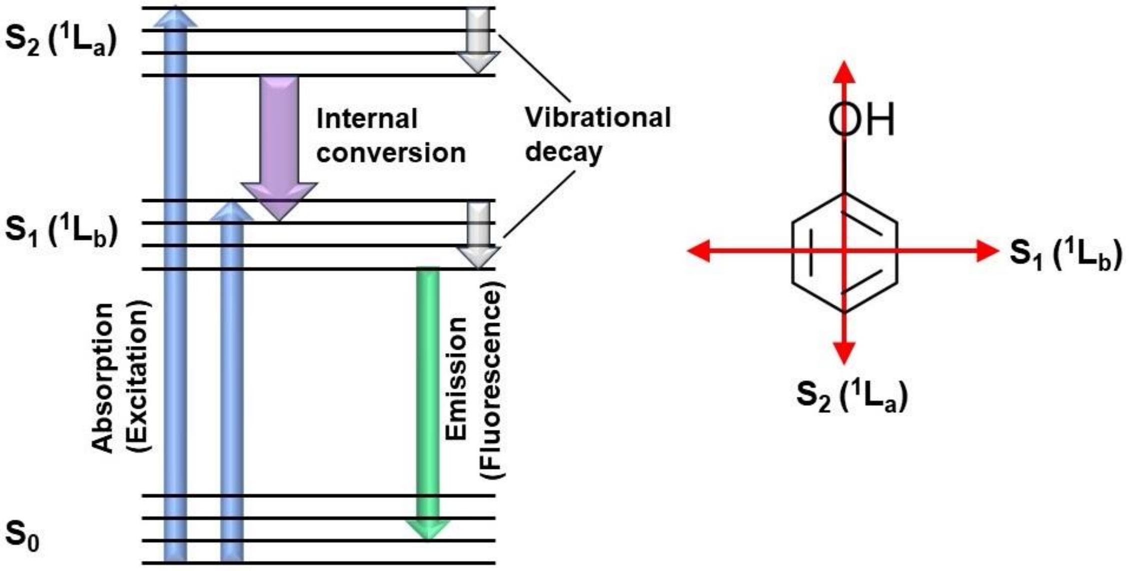

Supplementary Figure 7. Left: Jabłoński diagram showing the quantized energy levels of the ground state (S_0) and of the first and second excited singlet states (S_1 or ^1L_b and S_2 or ^1L_a). Blue upward arrows show ground-to-excited state transitions. The purple downward arrow shows the nonradiative conversion from a higher-energy to a lower-energy excited state. The grey downward arrows show nonradiative transitions to a lower vibrational energy level within the same excited singlet state, and the green downward arrow shows emission of fluorescence from the lowest energy level of the first excited state to an arbitrary vibrational energy level of the ground state. Right: Schematic presentation of the mutually orthogonal orientations of the S_1 (^1L_b) and S_2 (^1L_a) transition dipoles in phenol.

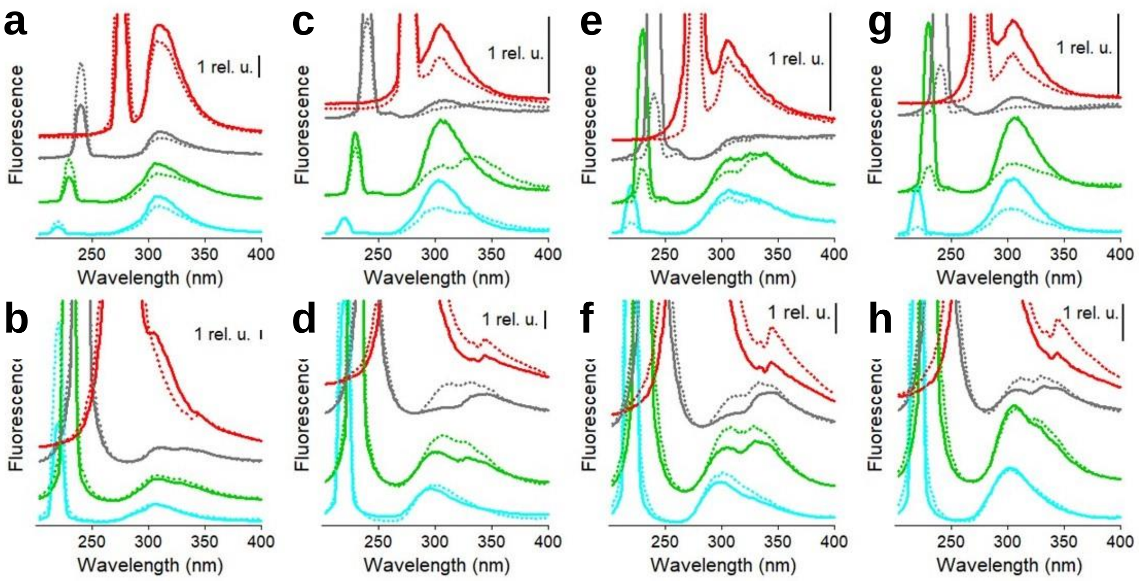

Supplementary Figure 8. Fluorescence spectra of Aβ_{1-42} (**a**, **b**), Aβ_{1-40} (**c**, **d**), AβpE$_{3-42}$ (**e**, **f**), and AβpE$_{3-40}$ (**g**, **h**) free in buffer (upper row) and in the presence of lipid vesicles (60 mol % POPC, 30 mol % POPG, 10 mol % cholesterol) (lower row). The excitation was at 220 nm (cyan), 230 nm (green), 240 nm (grey), and 275 nm (red). Dotted lines indicate that the samples have been extruded through 100 nm pore-size polycarbonate membranes. Peptide and total lipid (when present) concentrations were 20 μM and 1 mM, respectively, the buffer was 25 mM NaCl + 25 mM Tris-HCl, pH 7.2, and the temperature was 20°C. Aβ_{1-42} produced a single emission band at 306-313 nm with λ_{ex} = 220-275 nm, before and after extrusion (**a**). Spectra of extruded Aβ_{1-40} displayed split emission with λ_{ex} = 220-240 nm, with a red-shifted component at 336-338 nm (**c**). Splitting also occurred for unextruded and extruded AβpE$_{3-42}$, although with a smaller red-shift effect compared to that in phosphate buffer (**e**) (e.g., at λ_{ex} = 220 nm, the red-shifted component was at 325 nm in Tris buffer vs. 340 nm in phosphate buffer, cf. Figure 3**h** and Supplementary figure 8**e**). Spectra of AβpE$_{3-40}$ were not split in Tris buffer (**g**). The presence of lipid membranes did not cause significant changes in fluorescence spectra (**b**, **d**, **f**, **h**), except that the spectra of extruded AβpE$_{3-40}$ showed splitting (**h**), indicating more solvent-exposed Tyr of this peptide. Three independent experiments have been conducted with similar results.

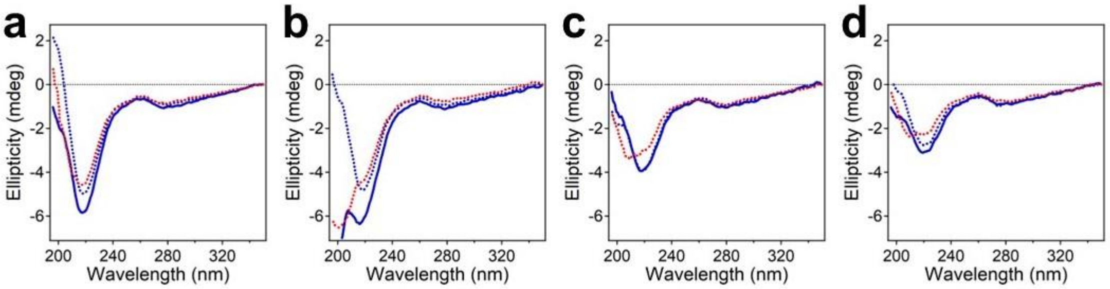

Supplementary Figure 9. Circular dichroism spectra of Aβ_{1-42} (**a**), Aβ_{1-40} (**b**), AβpE$_{3-42}$ (**c**), and AβpE$_{3-40}$ (**d**) in the absence (blue lines) and presence (red lines) of lipid vesicles composed of 60 mol % POPC, 30 mol % POPG, 10 mol % cholesterol. Dotted lines indicate that the samples have been extruded through 100 nm pore-size polycarbonate membranes. Peptide and total lipid (when present) concentrations were 20 μM and 1 mM, respectively, the buffer was 25 mM NaCl + 25 mM Tris-HCl, pH 7.2, and the temperature was 20°C. Three independent experiments have been conducted with similar results.

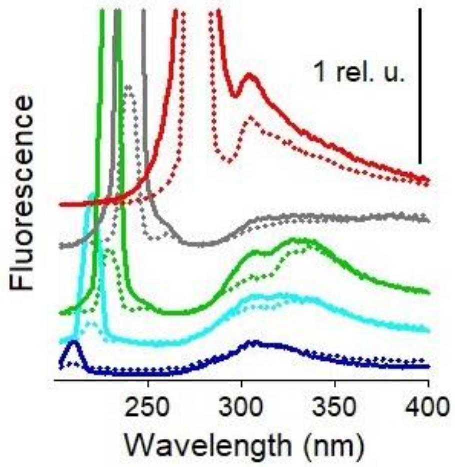

Supplementary Figure 10. Fluorescence spectra of AβpE$_{3-42}$ at 20 μM in 25 mM NaCl at 20°C. The excitation was at 210 nm (blue), 220 nm (cyan), 230 nm (green), 240 nm (grey), and 275 nm (red). Dotted lines indicate that the sample has been extruded through 100 nm pore-size polycarbonate membranes. The pH of the unbuffered solution was initially 8.4, then gradually decreased to 7.24 in ~ 30 min, apparently due to atmospheric CO_2, and that solution was used to suspend the peptide. Three independent experiments have been conducted with similar results.

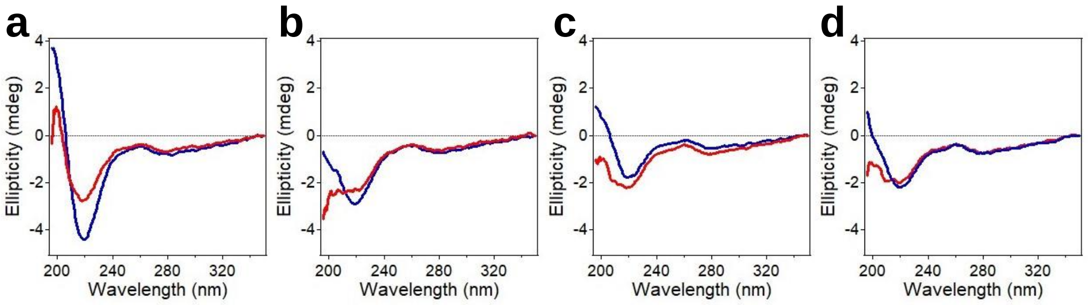

Supplementary Figure 11. Circular dichroism spectra of $A\beta_{1-42}$ (**a**), $A\beta_{1-40}$ (**b**), $A\beta pE_{3-42}$ (**c**), and $A\beta pE_{3-40}$ (**d**) at 37°C in the absence (blue lines) and presence (red lines) of lipid vesicles composed of 60 mol % POPC, 30 mol % POPG, 10 mol % cholesterol. All samples have been extruded through 100 nm pore-size polycarbonate membranes. Peptide and total lipid (when present) concentrations were 20 μM and 1 mM, respectively, the buffer was 25 mM NaCl + 25 mM Na,K-phosphate, pH 7.2. CD spectra show slightly more β-sheet content for $A\beta_{1-42}$ at 37°C (**a**). $A\beta_{1-40}$ formed β-sheet structure in buffer and the presence of vesicles caused unordered structure (**b**). $A\beta pE_{3-42}$ and $A\beta pE_{3-40}$ without vesicles adopted β-sheet structure with a minimum at 219 nm and the lipid induced partial α-helix with a shoulder at 207-210 nm (**c**, **d**). Three independent experiments have been conducted with similar results.

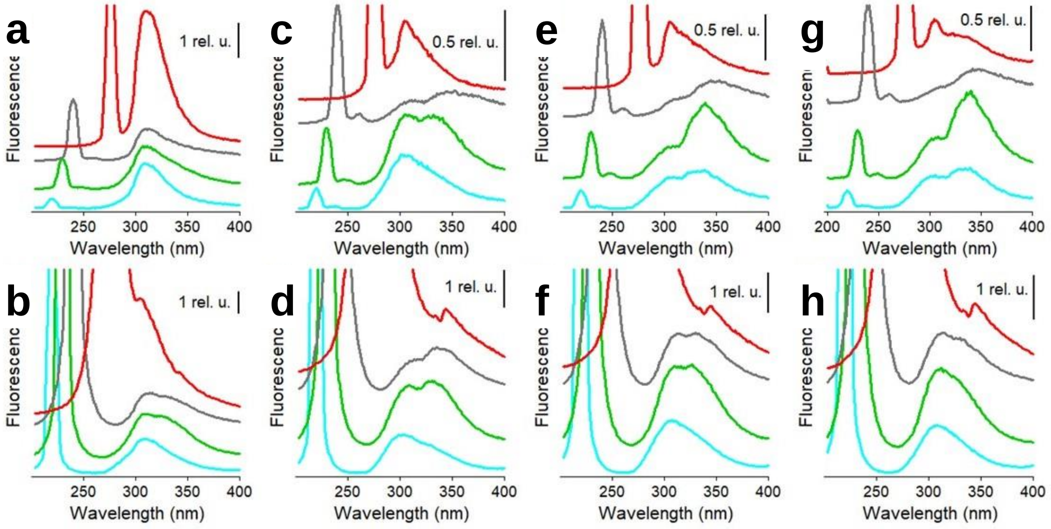

Supplementary Figure 12. Fluorescence spectra of $A\beta_{1-42}$ (**a**, **b**), $A\beta_{1-40}$ (**c**, **d**), $A\beta pE_{3-42}$ (**e**, **f**), and $A\beta pE_{3-40}$ (**g**, **h**) at 37°C in buffer (upper row) and in the presence of lipid vesicles (POPC:POPG:cholesterol = 6:3:1 molar ratio, lower row) with λ_{ex} = 220 nm (cyan), 230 nm (green), 240 nm (grey), and 275 nm (red). All samples have been extruded through 100 nm pore-size polycarbonate membranes. Peptide and total lipid (when present) concentrations were 20 μM and 1 mM, respectively, the buffer was 25 mM NaCl + 25 mM Na,K-phosphate, pH 7.2. Spectra of $A\beta_{1-42}$ are not split, those of $A\beta_{1-40}$ are split at λ_{ex} = 230 and 240 nm and the presence of lipid does not prevent it. The pyroglutamylated peptides produce split spectra at λ_{ex} = 220-240 nm, with the red-shifted component in the 332-352 nm region, and lipid vesicles protect these peptides from the solvent reflected in weakening or elimination of the red-shifted component. Three independent experiments have been conducted with similar results.

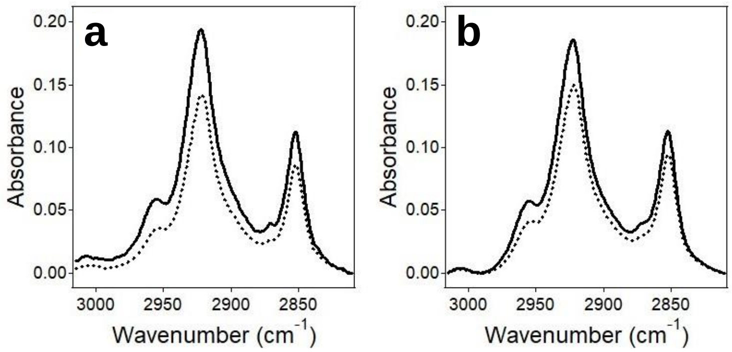

Supplementary Figure 13. (**a**): ATR-FTIR spectra of a supported lipid multilayer composed of 60 mol % POPC, 30 mol % POPG, and 10 mol % cholesterol, hydrated by a D_2O-based buffer of 25 mM NaCl, 25 mM Na,K-phosphate, pD 7.2, at parallel (solid) and perpendicular (dotted) polarizations of the incident light with respect to the plane of incidence. (**b**): Same with reconstituted peptide Aβ_{1-42} at peptide/lipid molar ratio of 1:50. Three independent experiments have been conducted with similar results. For all-*trans* hydrocarbon chains oriented strictly perpendicular to the Ge plate, a limiting value of lipid order parameter S_L = 1 is expected, and for totally unordered chains, S_L = 0. Taking into account that both POPC and POPG contain a double bond in their *sn*-2 acyl chains, which adopts *cis* configuration and thereby causes significant orientational disorder, an order parameter of 0.44 indicates reasonably well-organized membranes. For comparison, the order parameter of POPC monolayers on Ge plate was 0.05, and that of dipalmitoyl-phosphatidylcholine, a lipid with saturated acyl chains, was 0.7 (3). In other studies on POPC/POPG supported membranes, S_L values have been reported in the range between 0.3 and 0.7. The wavenumbers of asymmetric and symmetric CH_2 stretching vibrations occurred around 2921 cm^{-1} and 2852 cm^{-1}. CH_2 stretching wavenumbers are sensitive to the physical state of lipids; e.g., the asymmetric vibrational wavenumber increases from ~2916 cm^{-1} to ~2924 cm^{-1} upon thermal transition from sold state to fluid state (3). Wavenumbers presented in Table S2 indicate a fluid-like state of supported membranes, which reasonably imitate the properties of biological membranes.

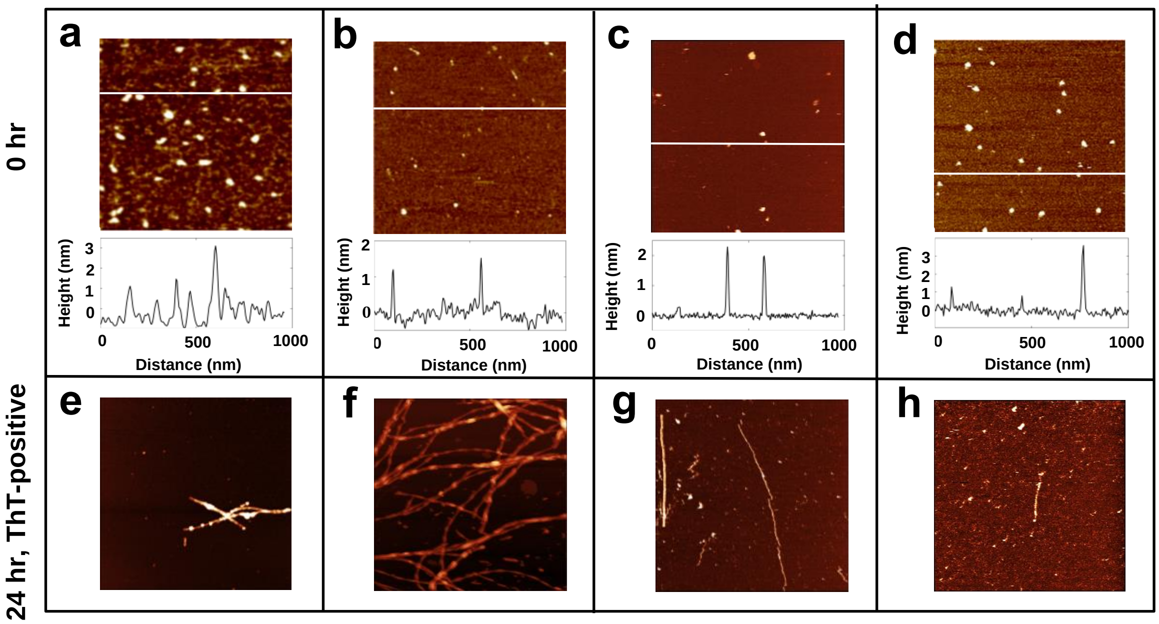

Supplementary Figure 14. AFM height images of Aβ_{1-42} (**a**, **e**), Aβ_{1-40} (**b**, **f**), AβpE$_{3-42}$ (**c**, **g**), and AβpE$_{3-40}$, (**d**, **h**) deposited on mica immediately after suspension in buffer containing 300 mM KCl and 10 mM HEPES, pH 7.4 (upper row) and incubated at 37°C for 24 h to induce fibrillogenesis (lower row). For panels **a**, **b**, **c**, **d**, the scan size is 1 µm × 1 µm, and the z-scale range is 0–5 nm. The white horizontal lines represent the cross-sections through the image corresponding to section profiles shown below respective images. (**e**): scan size 5 µm × 5 µm, z-scale 0–19 nm; (**f**): scan size 2 µm × 2 µm, z-scale 0–38 nm; (**g**): scan size 3 µm × 3 µm, z-scale 0–6 nm; (**h**): scan size 5 µm × 5 µm, z-scale 0–6 nm. Three independent experiments have been conducted with similar results.

Supplementary Table 1: Dichroic ratios (*R*) and average orientational angles for β-sheet ⟨β⟩ and α-helical components ⟨θ⟩ of the peptides in lipid membranes. The mean ± standard deviation values have been determined from three independent experiments.

	R_β	⟨β⟩ (°)		R_α	⟨θ⟩ (°)
		γ = 0°	γ =20°		
$A\beta_{1-42}$	1.681±0.081	30.9±1.1	30.0±1.40	1.701±0.076	65.3±3.3
$A\beta_{1-40}$	1.974±0.186	34.8±2.2	34.7±2.67	1.855±0.092	59.4±3.1
$A\beta pE_{3-42}$	2.234±0.138	37.8±1.4	38.3±1.71	2.221±0.073<br>2.112±0.070[a)]	48.9±1.8<br>51.7±1.8 [a)]
$A\beta pE_{3-40}$	1.754±0.124	32.0±1.8	31.3±2.19	1.919±0.086<br>1.963±0.130 [a]	57.3±2.7<br>56.0±3.9 [a)]

a) These spectra display components that can be assigned to α_{II}-helix, see Table 1 in the main text for their wavenumbers.

Supplementary Table 2: Lipid acyl chain dichroic ratios (R_L), order parameters (S_L), and peak wavenumbers of asymmetric and symmetric vibrations of methylene groups (ν_{as}, ν_{sym}) in the absence and presence of four Aβ peptides. The range of wavenumbers and mean ± standard deviation values have been determined from three independent experiments.

	Plane lipid	$A\beta_{1-42}$	$A\beta_{1-40}$	$A\beta pE_{3-42}$	$A\beta pE_{3-40}$
R_L [a)]	1.382±0.063	1.265±0.049	1.441±0.099	1.391±0.052	1.461±0.052
S_L	0.439±0.054	0.545±0.047	0.390±0.082	0.431±0.044	0.372±0.042
ν_{as} (cm^{-1})[b)]	2922.0-2921.1	2923.0-2922.0	2924.0-2923.0	2923.0-2922.0	2924.0-2923.0
ν_{sym} (cm^{-1})[b)]	2852.6-2851.6	2852.6-2851.6	2853.6-2852.6	2852.6	2852.6

a) Dichroic ratios based on total areas between 3015 cm^{-1} and 2810 cm^{-1}.
b) Wavenumbers at perpendicular polarization are lower by ~1 cm^{-1} compared to those at parallel polarization.

Supplementary Table 3. Channel length (*l*) and inner diameter (*d*) values corresponding to the measured single channel conductance values (g_{ch}) and structural parameters, simulated through Eq. 1. N_β is the number of amino acid residues in β-sheet conformation, β is the tilt angle of β-strands relative to the barrel axis (or membrane normal), ρ is the resistivity of KCl solution inside the channel.

Peptide	N_β	β (deg.)	*l* (nm)	g_{ch} (pS)	*d* (nm)	
					ρ = 0.08 Ωm	ρ = 0.4 Ωm
$A\beta_{1-42}$	19	30.5	5.7	100	0.245	0.559
$A\beta_{1-40}$	7	34.8	2.0	100<br>340	0.147<br>0.277	0.340<br>0.660
$A\beta pE_{3-42}$	14	38.0	3.84	150<br>300	0.248<br>0.355	0.572<br>0.828
$A\beta pE_{3-40}$	10	31.6	2.96	200<br>400<br>640<br>1040	0.254<br>0.364<br>0.466<br>0.603	0.591<br>0.861<br>1.118<br>1.477

Supplementary Methods

All four peptides were purchased from Innovagen (Lund, Sweden) in lyophilized form and were ≥ 98% pure. The lipids, 1-palmitoyl-2-oleoyl-phosphatidylcholine (POPC), 1-palmitoyl-2-oleoyl-phosphatidyglycerol (POPG) and cholesterol were from Avanti Polar Lipids (Alabaster, AL). Other chemicals such as salts, buffers, solvents were purchased from Sigma-Aldrich (St. Louis, MO) or Thermo Fisher Scientific (Waltham, MA). Quartz cuvettes for circular dichroism (CD) measurements were from Starna Calls, Inc. (Atascadero, CA). CaF_2 windows and other accessories for FTIR studies were from Buck Scientific (East Norwalk, CT) or Harrick Scientific Products Inc. (Pleasantville, New York). D_2O (99.8% pure) was from Cambridge Isotope Laboratories (Andover, MA).

Voltage Clamp Electrophysiology Experiments. For voltage clamp experiments, chloroform solutions of POPC, POPG, and cholesterol were combined at 6:3:1 molar into in a glass vial and the solvent was evaporated by desiccation. The dried lipids were dissolved in *n*-decane to a final concentration of 40-50 mg/mL (1 mg total mass of lipids). The bilayers were formed by the "painting" method, i.e. by applying the *n*-decane solution of the lipids to a 250 μm-wide aperture in a septum separating two chambers of a Derlin cuvette. After drying for 4 hours, 1 mL of the aqueous buffer (1 M KCl +10 mM HEPES, pH 7.4) was slowly added to each chamber so the aperture with the lipid film was submerged and the bilayer was allowed to form spontaneously, which took around 10 minutes. To facilitate bilayer formation by thinning of the lipid film, a glass-blob-terminated Pasteur pipette was used to gently brush over the aperture. Bilayer formation was monitored using real-time capacitance measurements. For all experiments, a 90-pF capacitance was set as the minimum threshold to ensure optimal bilayer formation. Generally, the capacitance of the bilayers varied between 100 pF and 180 pF due to variable bilayer area because of remaining bulk lipid solution along the aperture perimeter. After the

bilayer was formed in the aperture, the stability was tested using a combination of intermittent capacitance measurements and linear voltage ramp in the -150 mV to +150 mV range.

The voltage clamp setup consisted of a high-gain electrophysiology amplifier with a resistive feedback headstage (Warner Instruments, BC-535) connected to a digitizer (Digidata 1440A) which in turn was connected to a workstation computer. All data were recorded with a sampling frequency of 10 kHz. An integrated 8-pole low pass Bessel filter was used to filter the data at 1.0 kHz bandwidth. For further filtering and noise reduction, the data were also collected in parallel with another Bessel filter (Warner Instruments, LPF-8) at a cutoff frequency of 60 Hz. For controlling and visualization of the signals in real-time, Clampex software was used (v10.6, Axon Instruments) and all the subsequent data analysis was carried out with Clampfit (v10.6, Axon Instruments, San Jose, CA). Conductance histograms were constructed using OriginPro 2023 (OriginLab).

The lyophilized peptide was dissolved in 1% NH_4OH, divided into aliquots and stored at -80^oC. An aliquot was thawed in an ice bath and buffer solution (1 M KCl +10 mM HEPES, pH 7.4) was added to dilute the peptide to the required concentration and the solubilized peptide was added to one of the wells (trans side) at the required final concentration. In most cases, electrophysiological activity was assessed at peptide final concentration of 100 nM. After about 5 min of peptide addition, bilayer hold voltages were ramped in 50 mV increments from -100 mV to +100 mV (the sign corresponds to the trans side). Current traces were recorded for several minutes at each hold voltage. After certain pattern of the electrical activity of the membrane was established, ion channel blocking by Zn^{2+} ions was assessed. A solution of $ZnCl_2$ was added to the trans side at final concentration of 10 mM or 20 mM and the current was subsequently recorded for additional several minutes. All buffer or salt solutions were freshly prepared and filtered through 0.22 μm PTFE filters before usage.

Electrophysiology Data Analysis. The recorded data files were transferred into the pClamp analysis environment and a manual baseline correction was performed in conjunction with offset removal. For analysis and visualization of the electrophysiological activity of the peptides at each holding voltage, 120-180 s snippets were selected, where the electrical activity was visually clear. Amplitude histograms of these snippets were generated to display the distribution of conductance values.

Channel diameter (*d*) was estimated using the formula (4,5):

$$d = \frac{\rho g_{ch}}{2}\left(1 + \sqrt{1 + \frac{16l}{\pi\rho g_{ch}}}\right) \quad (1)$$

where ρ is the resistivity of the salt solution inside the channel, g_{ch} is the measured single channel conductance, and *l* is the channel length. As the best single channel forming peptide(s) studied in this work have significant β-sheet structures with β-strands tilted at β = 30-40 degrees relative to the membrane normal, we have estimated the channel length assuming a β-barrel-like channel structure, i.e., $l = aN_\beta\cos\beta$, where *a* is the displacement along the β-strand axis per amino acid residue (*a* = 3.48 Å) and N_β is the number of amino acids in β-sheet conformation (6). Determination of the value of ρ is problematic as the properties of both the solvent (water) and the solute (ions) in a narrow channel are different from those in the bulk phase. Confinement reduces the motional freedom and the polarizability of water molecules, resulting in a reduced local dielectric constant and thereby creating an energy barrier for the ions through the Born factor. Reduced motional freedom also increases the local viscosity and decreases the diffusion

coefficient of the ions. Thus, neither the bulk ion concentration nor the bulk diffusion coefficient or the dielectric constant are operative within the channel. Since these effects depend on the pore radius (more accentuated for narrower pores), their theoretical estimation is overly complex. A practical solution has been offered that uses an empirical correction factor to match the measured and simulated conductances for pores with known structures (7,8). The values of the correction factor, which indicates how many times the effective solvent resistivity increases inside the pore compared to the bulk phase, varied in a range but were clustered around 5.0 (5.0 for *E. coli* OmpF porin and 5.6 for gramicidin A channel) (7). Hence, we have estimated the channel diameter using both the bulk resistivity of 1 M KCl, i.e., $\rho = 0.08$ Ωm, and the corrected value, $\rho = 0.4$ Ωm.

AFM Imaging. Membrane organization and the morphology of the Aβ peptides were probed by atomic force microscopy (AFM) imaging on peptides directly deposited on mica as well as reconstituted in lipid bilayers. For AFM imaging on mica, the peptide stocks were diluted to 1 µM final concentration in buffer containing 300 mM KCl and 10 mM HEPES (pH 7.4) and vortexed for 30 s, then 20 µL of the peptide solution was added to a freshly cleaved mica disk. The sample was incubated at room temperature for ~10 minutes, washed 3 times with the same (imaging) buffer, and scanned. For AFM imaging with lipids, dried lipids were hydrated by the imaging buffer supplemented with 3 mM $CaCl_2$ at room temperature for about 20 minutes. The hydrated lipid sample was then bath-sonicated for 15 minutes to form unilamellar vesicles. After formation of vesicles, the peptide was added to the vial at 1:1000 or 1:500 peptide-to-lipid molar ratio and incubated for 10 minutes at room temperature. For better incorporation of peptides into vesicle membranes, the peptide/lipid sample was briefly bath-sonicated for 60-75 s. The sample consisting of proteoliposomes was then drop-casted on the freshly cleaved mica surface and incubated for 20 minutes at room temperature, washed 3 times with the imaging buffer and the formed supported lipid bilayers were imaged in the same buffer. All AFM imaging was performed on a Multimode AFM (Bruker) controlled by a Nanoscope V controller. All images were collected in PeakForce Tapping mode with SNL-10 cantilevers (Bruker) of spring constant in the range of 0.1-0.3 N/m and a nominal tip diameter of 2 nm. Fresh cantilevers were used in every imaging session and among different peptides.

AFM Data Analysis. All collected images were analyzed using Nanoscope Analysis v1.5 software or the SPM analysis package Gwyddion. Briefly, images were line flattened and color scale adjusted. For generating particle height histograms, we used the "Particle Analysis" feature in Nanoscope Analysis, which categorizes particles based on height thresholding.

CD and Fluorescence. For CD and fluorescence experiments, the lyophilized lipids were dissolved in chloroform at 2.00 mM concentration and the peptides were dissolved in hexafluoroisopropanol (HFIP) at 200 µM concentration as stock solutions. Peptide samples were prepared by placing 50 µL of 200 µM peptide solution in HFIP in a glass vial, drying by 1-h desiccation, adding 500 µL of the buffer and vortexing for 5 minutes. To prepare the proteoliposomes, a mixture of 150 µL of 2 mM POPC, 75 µL of 2 mM POPG, 25 µL of 2 mM cholesterol, and 50 µL of 200 µM peptide were combined in a glass vial and desiccated for 1 hour. Then 500 µL of buffer was added and vortexed for 5 minutes, resulting in total lipid concentration of 1.00 mM at POPC:POPG:cholesterol molar ratio of 6:3:1 + 20 µM peptide, corresponding to1:50 peptide-to-total-lipid molar ratio. The buffer was either 25 mM NaCl + 25 mM Na,K-phosphate (pH 7.2) or 25 mM NaCl + 25 mM Tris-HCl (pH 7.2). The buffer solutions

were filtered with 0.22 μm filter-syringes and were kept at 4°C. When needed, the peptide or proteoliposome samples were extruded through 100 nm pore-size polycarbonate membranes (15 passages) using an Avanti Polar Lipids mini extruder.

CD and fluorescence spectra were measured using a J-810 spectropolarimeter equipped with a fluorescence attachment and a temperature controller and operated by the Spectra Manager 2.8 software (Jasco, Tokyo, Japan). Ten scans were averaged for the CD spectra and 3 scans for the fluorescence spectra.

Attenuated Total Reflection Fourier Transform Infrared Spectroscopy. For attenuated total reflection Fourier transform infrared (ART-FTIR) experiments, 18.75 µL of 20 mM POPC, 9.375 µL of 20 mM POPG, 3.125 µL of 20 mM cholesterol, dissolved in chloroform, and 62.5 µL of 200 μM peptide dissolved in HFIP were mixed in a vial and spread over the surface of a germanium plate (5 cm × 2 cm × 0.1 cm, cut at the 2 cm edges at a 45° bevel angle). The sample was air-dried, then desiccated for 1 hour before assembly into a flow-through ATR sample cell (Buck Scientific, East Norwalk, CT). One mL of the buffer (25 mM NaCl + 25 mM Na,K-phosphate in D_2O, pD 7.2) was injected into the cell to hydrate the peptide-lipid sample, which, according to appropriate calculations, forms a 700-800 nm-thick multilayer. The cell was mounted onto a four-mirror ATR sample holder (Buck Scientific) and placed in a Vector-22 FTIR spectrometer (Bruker, Billerica, MA, USA) equipped with a liquid nitrogen-cooled Hg/Cd/Te detector and an aluminum-grid-on-KRS-5 polarizer (Specac, Newmarket, Suffolk, UK), operated by OPUS 8.7 SP2. Under these settings, the infrared light enters the germanium plate at a 90° incidence angle relative to the 2 cm beveled edge and undergoes a series of internal reflections at 45° incidence angle before it exits the plate and travels to the detector. At each internal reflection, an evanescent wave is created that is absorbed by the sample deposited on the plate. Transmission spectra were recorded between 4000 cm^{-1} and 400 cm^{-1} at parallel (‖) and perpendicular (⊥) polarizations of the incident light (when the electric vector of the plane-polarized light is coplanar or perpendicular to the plane of incidence), and 300 scans were collected per spectrum. Transmission spectra of the blank buffer were measured separately and used as reference to obtain the respective absorbance spectra.

ATR-FTIR Data Analysis. The ART-FTIR spectra were used to determine the secondary structure and orientation of the peptides relative to the membrane and the structural order of the lipid acyl chains. A peak-fitting procedure was applied, using the GRAMS/AI™ Spectroscopy software (Galactic Industries Corp.) to identify the spectral components in the lipid C=O and peptide amide I regions. The overall areas of each component were then determined using both ‖ and ⊥ spectra as follows: $a_i = a_{i,||} + Ga_{i\perp}$, where a_i is the area of the ith component and G is a scaling factor, which for present experimental conditions is $G = 1.44$ (9). The fractions of α-helix, β-sheet, unordered (ρ), and "other" structures were calculated using the amide I areas of respective components (a_i) and their integrated molar absorptivities (B_i) as described earlier (9):

$$f_i = \frac{a_i}{B_i\left(\frac{a_\alpha}{B_\alpha}+\frac{a_\beta}{B_\beta}+\frac{a_\rho}{B_\rho}+\frac{a_{other}}{B_{other}}\right)} \quad (2)$$

Previously estimated values for B have been used: $B_\alpha = 7.6\times10^7$ cm/mol, $B_\beta = 6.2\times10^7$ cm/mol, $B_\rho = 4.5\times10^7$ cm/mol (10). For "other" structures, which may involve mainly various types of turns and other conformations, an average value of $B_{other} = 5.5\times10^7$ cm/mol has been used. It should be noted that these are average B values reported for proteins in H_2O-based buffer (10)

and are larger by 20-70% compared to previously reported values estimated for amide-deuterated proteins in D_2O-based buffer (11,12). Venyaminov and Kalnin (10) report that *B* values for unordered and β-sheet structures are similar in H_2O- and D_2O-based buffers whereas for α-helix $B_\alpha = B_\beta$ in H_2O and $B_\alpha = B_\rho$ in D_2O (10). In our experiments, the peptide was embedded in lipid layers, desiccated, followed by injection of the D_2O-based buffer and recording of spectra within the next hour. Since the amide-deuteration of membrane-embedded proteins proceeds with a time constant exceeding 2 hours (13), the peptides were only partially deuterated and hence the use of *B* values corresponding to the H-form is justified. On the other hand, as seen from Eq. (2), the ratio of *B* values rather than the absolute values determine the secondary structure fractions. Thus, the present evaluation of the secondary structure fractions is reasonable within the available data on molar absorptivities. The assignment of amide I components to secondary structure types was done according to established wavenumber ranges for various protein structures, i.e., turn structures: 1705-1661 cm^{-1}, α-helix: 1660-1646 cm^{-1}, unordered: 1645-1638 cm^{-1}, β-sheet: 1637-1623 cm^{-1} (9). Amide I components below 1620 cm^{-1} were assigned to side chains.

The orientational order parameter of α-helical structure was determined using the formula (9):

$$\boldsymbol{S} = \frac{\boldsymbol{2C}}{(\boldsymbol{3cos^2\alpha - 1})(\boldsymbol{C - 3E_z^2})} \quad (3)$$

In Eq. (3), $C = E_x^2 - RE_y^2 + E_z^2$, α is the angle between the transition dipole moment and the molecular axis (for α-helix, $\alpha = 39^o \pm 1^o$), $E_x = 1.399$, $E_y = 1.514$, and $E_z = 1.621$ are the electric vector components of the evanescent wave, and *R* is the dichroic ratio, i.e. $R = a_{\parallel}/a_{\perp}$. Values of *R* for α-helix have been determined using the α-helical component areas and used to calculate the helical order parameter. The time- and ensemble-averaged angle of orientation of helical axis relative to the membrane normal, ⟨θ⟩, was calculated from the order parameter through:

$$\langle cos\theta \rangle = \sqrt{\frac{2S+1}{3}} \quad (4)$$

The orientation of β-strands can be determined from β-sheet dichroic ratio (in the region 1637-1623 cm^{-1}) of a protein or a peptide that adopts a structure with a rotational axis of symmetry, such as a β-barrel. Then, the following relationship holds (9):

$$\boldsymbol{\frac{1}{2}(3cos^2\delta - 1)} = \frac{\boldsymbol{2C}}{(\boldsymbol{3cos^2\gamma - 1})(\boldsymbol{C - 3E_z^2})} \quad (5)$$

In Eq. (5), δ is the angle of the transition dipole moment of β-strands with respect to the central axis, and γ is the tilt angle of the central axis of the barrel structure with respect to the membrane normal. The amide I transition dipole is oriented perpendicular to the β-strand axis, which yields the following relationship between angle δ and the angle β of orientation of the strand axis relative to the barrel's central axis:

$$\boldsymbol{\delta = \frac{\pi}{2} - \beta} \quad (6)$$

The angle β for the barrel structure can be determined through Eqs. (5) and (6) using the measured dichroic ratio for the β-sheet component of the amide I spectrum if the angle γ is known. In a situation when γ is not known, a practically useful approach is to assume conceivable values, such as γ = 0 degrees or γ = 20 degrees, and to determine the orientations of strands in a β-barrel. This approach has been adopted in this work.

The order parameter of lipid acyl chains in supported membranes was determined by analysis of the ATR-FTIR spectral region corresponding to methylene (CH_2) stretching vibrations. The CH_2 groups of lipid acyl chains undergo asymmetric and symmetric stretching vibrations and generate absorbance bands at wavenumbers around 2920 cm^{-1} and 2850 cm^{-1}, respectively (3). For a hydrocarbon chain in all-trans configuration, the transition dipole moment of CH_2 vibrations is oriented perpendicular to the chain axis. Therefore, the order parameter of lipid acyl chains can be determined through Eq. (3) using $\alpha = 90^{o}$. Dichroic ratios have been determined as $R_L = a_{L,||} / a_{L,\perp}$, where $a_{L,||}$ and $a_{L,\perp}$ are the total areas of lipid CH_2 absorbance bands from 3015 cm^{-1} to 2810 cm^{-1} at ‖ and ⊥ polarizations of the incident light, respectively. The statistical analysis of the S_L values was conducted using an online *t* test calculator (https://www.graphpad.com/quickcalcs/ttest1/?format=SD).

Supplementary References

1. Lakowicz, J. R. Principles of Fluorescence Spectroscopy, 2nd ed. (Kluwer Academic/Plenum Publishers, 1999).
2. Antosiewicz, J. M. & Shugar, D. UV-Vis spectroscopy of tyrosine side-groups in studies of protein structure. Part 1: basic principles and properties of tyrosine chromophore. *Biophys Rev*. **8(2),** 151−161 (2016).
3. Tatulian, S. A. Attenuated total reflection Fourier transform infrared spectroscopy: A method of choice for studying membrane proteins and lipids. *Biochemistry* **42,** 11898−11907 (2003).
4. Bode D. C., Baker. M. D. & Viles, J. H. Ion Channel Formation by Amyloid-β42 Oligomers but Not Amyloid-β40 in Cellular Membranes. *J Biol Chem.* **292(4),** 1404−1413 (2017).
5. Lee, J., Gillman, A. L., Jang, H., Ramachandran, S., Kagan, B. L., Nussinov, R. & Teran Arce F. Role of the fast kinetics of pyroglutamate-modified amyloid-β oligomers in membrane binding and membrane permeability. *Biochemistry* **53(28),** 4704−4714 (2014).
6. Kandel, N., Matos, J. O. & Tatulian, S. A. Structure of amyloid β_{25-35} in lipid environment and cholesterol-dependent membrane pore formation. *Sci Rep.* **9(1),** 2689 (2019).
7. Smart, O. S., Breed, J., Smith, G. R. & Sansom, M. S. A novel method for structure-based prediction of ion channel conductance properties. *Biophys J.* **72(3),** 1109−1126 (1997).
8. Tieleman, D. P. & Berendsen, H. J. A molecular dynamics study of the pores formed by *Escherichia coli* OmpF porin in a fully hydrated palmitoyloleoylphosphatidylcholine bilayer. *Biophys J.* **74(6),** 2786−2801 (1998).
9. Tatulian, S. A. FTIR analysis of proteins and protein-membrane interactions. *Methods Mol. Biol.* **2003,** 281−325 (2019).
10. Venyaminov, S. Y. & Kalnin, N. N. Quantitative IR spectrophotometry of peptide compounds in water (H_2O) solutions. II. Amide absorption bands of polypeptides and fibrous proteins in α-, β-, and random coil conformations. *Biopolymers* **30,** 1259−1271 (1990).
11. Chirgadze, Y. N., Shestopalov, B. V. & Venyaminov, S. Y. Intensities and other spectral

parameters of infrared amide bands of polypeptides in the beta- and random forms. *Biopolymers* **12(6),** 1337−1351 (1973).
12. Chirgadze, Y. N. & Brazhnikov, E. V. Intensities and other spectral parameters of infrared amide bands of polypeptides in the alpha-helical form. *Biopolymers* **13(9),** 1701−1712 (1974).
13. Tatulian, S. A., Cortes, D. M. & Perozo, E. Structural dynamics of the *Streptomyces lividans* K^+ channel (SKC1): secondary structure characterization from FTIR spectroscopy. *FEBS Lett.* **423(2),** 205−212 (1998).
