# Supplementary material for: The structure of tyrosine-10 favors ionic conductance of Alzheimer’s disease-associated full-length amyloid-β channels
Source: Nat Commun. 2024 Feb 13;15:1296. doi: 10.1038/s41467-023-43821-y (PMC10864385; doi:10.1038/s41467-023-43821-y)
Supplement: Supplementary file 3 — Reporting Summary [file 41467_2023_43821_MOESM3_ESM.pdf]

nature portfolio

Corresponding author(s): Suren A. Tatulian

Last updated by author(s): 11 November, 2023

Reporting Summary

Nature Portfolio wishes to improve the reproducibility of the work that we publish. This form provides structure for consistency and transparency in reporting. For further information on Nature Portfolio policies, see our Editorial Policies and the Editorial Policy Checklist.

Statistics

For all statistical analyses, confirm that the following items are present in the figure legend, table legend, main text, or Methods section.

n/a	Confirmed	
☐	☒	The exact sample size (*n*) for each experimental group/condition, given as a discrete number and unit of measurement
☐	☒	A statement on whether measurements were taken from distinct samples or whether the same sample was measured repeatedly
☐	☒	The statistical test(s) used AND whether they are one- or two-sided<br>*Only common tests should be described solely by name; describe more complex techniques in the Methods section.*
☒	☐	A description of all covariates tested
☒	☐	A description of any assumptions or corrections, such as tests of normality and adjustment for multiple comparisons
☐	☒	A full description of the statistical parameters including central tendency (e.g. means) or other basic estimates (e.g. regression coefficient) AND variation (e.g. standard deviation) or associated estimates of uncertainty (e.g. confidence intervals)
☒	☐	For null hypothesis testing, the test statistic (e.g. *F*, *t*, *r*) with confidence intervals, effect sizes, degrees of freedom and *P* value noted<br>*Give P values as exact values whenever suitable.*
☒	☐	For Bayesian analysis, information on the choice of priors and Markov chain Monte Carlo settings
☒	☐	For hierarchical and complex designs, identification of the appropriate level for tests and full reporting of outcomes
☒	☐	Estimates of effect sizes (e.g. Cohen's *d*, Pearson's *r*), indicating how they were calculated

Our web collection on statistics for biologists contains articles on many of the points above.

Software and code

Policy information about availability of computer code

Data collection	OPUS 8.7 SP2 (Bruker) for FTIR data collection<br>Spectra Manager 2.8 (Jasco) for circular dichroism and fluorescence data collection<br>Nanoscope 9.1 (Bruker) software for AFM control and data acquisition<br>Clampex 10.7 (Axon Instruments) for collection of voltage clamp electrophysiology signals
Data analysis	GRAMS/AI™ Spectroscopy software (Galactic Industries Corp.) for FTIR data analysis<br>IGOR Pro 5.03 (WaveMetrics) for circular dichroism and fluorescence data analysis<br>Nanoscope analysis 1.5 (Bruker) and Gwyddion (open source) softwares for AFM image postprocessing<br>Clampfit 10.7 (Axon Instruments) for postprocessing of electrophysiology data<br>Matlab (Mathworks) for histogram plot generation<br>OriginPro 2023 (OriginLab) for histogram plot generation

For manuscripts utilizing custom algorithms or software that are central to the research but not yet described in published literature, software must be made available to editors and reviewers. We strongly encourage code deposition in a community repository (e.g. GitHub). See the Nature Portfolio guidelines for submitting code & software for further information.

Data

Policy information about availability of data

All manuscripts must include a data availability statement. This statement should provide the following information, where applicable:

- Accession codes, unique identifiers, or web links for publicly available datasets
- A description of any restrictions on data availability
- For clinical datasets or third party data, please ensure that the statement adheres to our policy

The data have not been made publicly available before manuscript acceptance.

Research involving human participants, their data, or biological material

Policy information about studies with human participants or human data. See also policy information about sex, gender (identity/presentation), and sexual orientation and race, ethnicity and racism.

Reporting on sex and gender	N/A
Reporting on race, ethnicity, or other socially relevant groupings	N/A
Population characteristics	N/A
Recruitment	N/A
Ethics oversight	N/A

Note that full information on the approval of the study protocol must also be provided in the manuscript.

Field-specific reporting

Please select the one below that is the best fit for your research. If you are not sure, read the appropriate sections before making your selection.

☒ Life sciences ☐ Behavioural & social sciences ☐ Ecological, evolutionary & environmental sciences

For a reference copy of the document with all sections, see nature.com/documents/nr-reporting-summary-flat.pdf

Life sciences study design

All studies must disclose on these points even when the disclosure is negative.

Sample size	All in vitro experiments have been done in triplicate to ensure reproducibility and to obtain reasonable standard deviations of numerical data, which is a standard approach for biophysical studies.
Data exclusions	No data were excluded
Replication	All data were reproducible provided the experimental conditions were kept unchanged.
Randomization	The experimental samples were randomly allocated into groups.
Blinding	Blinding the investigators was not necessary and is not typical for this kind of research. However, different pieces of data obtained in one laboratory were not disclosed to the other laboratory before the data from the latter were reported.

Reporting for specific materials, systems and methods

We require information from authors about some types of materials, experimental systems and methods used in many studies. Here, indicate whether each material, system or method listed is relevant to your study. If you are not sure if a list item applies to your research, read the appropriate section before selecting a response.

Materials & experimental systems

n/a	Involved in the study
☒	☐ Antibodies
☒	☐ Eukaryotic cell lines
☒	☐ Palaeontology and archaeology
☒	☐ Animals and other organisms
☒	☐ Clinical data
☒	☐ Dual use research of concern
☒	☐ Plants

Methods

n/a	Involved in the study
☒	☐ ChIP-seq
☒	☐ Flow cytometry
☒	☐ MRI-based neuroimaging
